# From Digenic to Monogenic Sex Determination in Insects: A Genetic Model Based on Imprinting and X Chromosome Elimination

**DOI:** 10.3390/genes16121478

**Published:** 2025-12-09

**Authors:** Lucas Sánchez

**Affiliations:** Departamento de Biotecnología, Centro de Investigaciones Biológicas “Margarita Salas”, The Spanish National Research Council, 28040 Madrid, Spain; lsanchez@cib.csic.es

**Keywords:** insects, sex determination, evolution, monogenic species, chromosome elimination, chromosome imprinting

## Abstract

**Background/Objectives:** In digenic populations, all females produce males and females in their offspring. Monogenic populations are composed of gynogenic (female-producing) and androgenic (male-producing) females. A theoretical population genetic model for evolution of digenic to monogenic populations is presented here. **Methods:** A controlling gene was associated with each of the four processes that characterise monogenic populations: (1) oogenesis is conventional, whereas spermatogenesis is unusual and it is characterised by the exclusive formation of X-bearing sperm (gene (*s*)), i.e., the paternal chromosomes are eliminated so that only the maternal ones are transmitted to the next generation; (2) the X chromosome that is eliminated in the zygote is the one inherited from the father (gene *r*); (3) an imprinting process occurs in the mother (gene *g*), which protects the maternally inherited X chromosome from elimination in the zygote and the whole maternal chromosome complement in spermatogenesis; (4) a maternal factor is produced during oogenesis (gene *e*), which inactivates the elimination factor [r] in the zygote, thus controlling the elimination of the paternal X chromosome. The sequences of emergence of the genes (*e s r g*) that transform a digenic population into a monogenic one were analysed. **Results:** The following evolutionary sequences were found: (1) the sequence (*r s e*) under dominant conditions of gene (*s*) and recessive conditions of gene (*r*); and (2) the sequences (*s r e*), (*r s e*), and (*e s r*) under recessive conditions of gene (*s*) and gene (*r*). It was also found that the process of genomic imprinting is a necessary condition for the generation of a monogenic population. Furthermore, a quantitative change in the interaction between the elimination factor and its maternal inhibitor modifies the genotypic formula of the monogenic state. **Conclusions:** The number and types of evolutionary transitions of a digenic to a monogenic population depends on the dominant or recessive characteristic of the newly emerging genes. The imprinting process must already be present in the digenic population from which the monogenic one evolves; otherwise, the population cannot reach the monogenic state.

## Simple Summary

As a rule, the sex of an individual is fixed at fertilisation, and the chromosomal constitution of the zygote is a direct consequence of the chromosomal constitution of the gametes. A theoretical evolutionary population genetic model is presented to explain the hypothesised evolutionary transition routes from the ancestral XX/X0 mechanism of sex determination to one in which all zygotes are initially equal and sex determination occurs by elimination of one X chromosome in the zygote. This mechanism has four characteristics to which a control gene is associated: (1) oogenesis is conventional, whereas spermatogenesis is characterised by the exclusive formation of X-bearing spermatozoa (gene *s*); (2) the eliminated X chromosome (gene *r*) is the one inherited from the father; (3) an imprinting process occurs in the mother (gene *g*), which protects the maternally inherited X chromosome from elimination; (4) during oogenesis, a maternal factor (gene *e*) is produced, which inactivates the elimination factor [r], thereby regulating the elimination of the X chromosome in the zygote. The sequence of emergence of the four genes (*e*, *s*, *r*, *g*) that transform a digenic population into a monogenic one composed of gynogenic females producing females and androgenic females producing males is analysed.

## 1. Introduction

The animal kingdom exhibits diverse mechanisms by which gender is determined [1,2]. This is especially evident in insects, where all known types of sex determination mechanisms are represented [3,4,5,6]. These mechanisms can be classified into three main categories according to the origin of the primary sex determination signal, which can be zygotic, maternal, or environmental.

**Figure 1:**
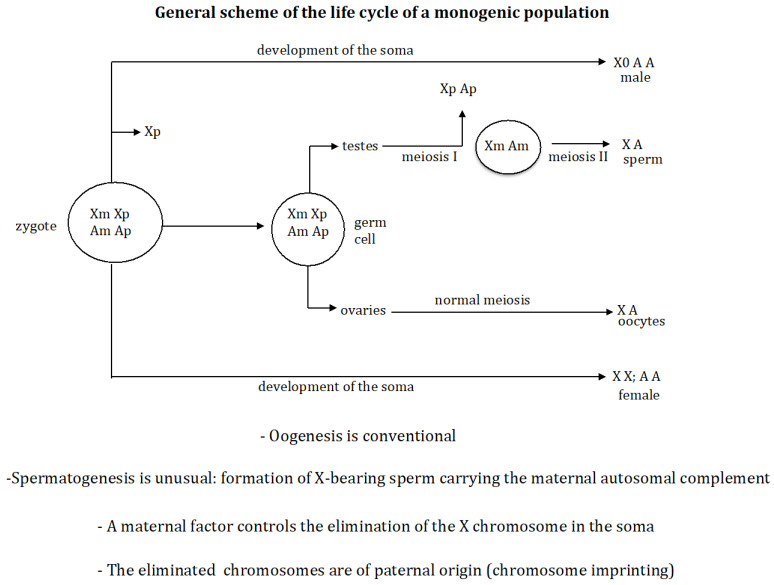
General scheme of the life cycle of a monogenic population. First, oogenesis is conventional whereas spermatogenesis is unusual, characterised by the *exclusive formation of X-bearing sperm*. Second, the chromosomes that are eliminated (or inactivated) are those *inherited from the father*. Third, an *imprinting process* occurs in the mother, which determines that the chromosomes to be eliminated or inactivated are of paternal origin. And fourth, in the cases studied, an unknown *maternal factor* is produced during oogenesis, which accumulates in the oocyte and then governs the elimination of the X chromosome in the developing zygote [4]. “X” stands for X chromosome and “A” stands for a set of autosomes; “m” and “p” represent the chromosomes of maternal and paternal origin, respectively.

There are two classes of species according to the type of offspring produced by the females: digenic and monogenic. The former are characterised by producing both males and females in their offspring, whilst the latter are characterised by the existence of two types of females in the population: gynogenic, which only produce females in their offspring, and androgenic, which only produce males in their offspring. As a general rule, the sex of an individual in digenic species is fixed at fertilisation, and the chromosomal constitution of the zygote is a direct consequence of the chromosomal constitution of the gametes. In monogenic species, however, the chromosomal differences that determine sex are produced by the elimination of chromosomes in the embryo. The monogenic mechanism that forms the sex-determining chromosomal signal that triggers the sexual fate of the embryo has four characteristics in common (Figure 1).

First, oogenesis is conventional whilst spermatogenesis is unusual, characterised by the *exclusive formation of X-bearing sperm*. This results in all zygotes having the same chromosome constitution. Second, the chromosomes that are eliminated are those *inherited from the father*. Third, an *imprinting process* occurs in the female, which determines that the chromosomes to be eliminated are of paternal origin. And fourth, in the cases studied, an unknown factor is produced during oogenesis that accumulates in the oocyte and then governs the elimination of the X chromosome in the developing zygote [3,4]. For example, in sciarid insects (fungal gnats), all zygotes start with the XXX constitution; the loss of one or two X chromosomes determines whether the zygote becomes XX (female) or X0 (male) [7,8]. In Cecydomyiidae insects (gall midges), all zygotes begin with the X_1×1×2×2_ constitution. If the embryo does not delete any X chromosomes, it remains as X_1_X_1_X_2_X_2_ and develops as female, whereas if two X chromosomes (X_1_X_2_) are deleted, the embryo becomes X_1_X_2_0 and develops as male [9,10,11]. A similar situation is found in Collembola insects (springtails), where zygotes start out with the XXXX constitution. If the embryo does not delete any X chromosome, it remains as XXXX and develops as a female, whereas if two X chromosomes are deleted, the embryo becomes XX0 and develops as a male [12,13,14].

Haig presented an evolutionary model for the sex determination mechanism of sciarids [15]. He proposed that this mechanism results from an intragenomic conflict between the effects of male-biased and female-biased sex ratios on genetic functions arising in the population. He suggested the following evolutionary scenario for sciarids. A driving X chromosome that gained a transmission advantage, causing a female-biased sex ratio, arose in the population. This produced a situation that was exploited by maternal autosomes to segregate with the X chromosome in spermatogenesis. The female-biased sex ratio was counteracted by maternally affected selection for genes causing the transformation of XX zygotes into X0 males by the loss of the paternally derived X chromosome, together with the transformation of some X chromosomes into germ-limited L chromosomes, producing a male-biased sex ratio. Finally, an X chromosome emerged that suppressed the effects of the L chromosomes. The studies mentioned did not explicitly consider gene emergence or the process of chromosomal imprinting that is involved in the sex determination process analysed here.

The monogenic mechanism that forms the sex-determining chromosomal signal that triggers the sexual fate of the embryo has four characteristics in common (Figure 1). First, oogenesis is conventional whilst spermatogenesis is unusual, characterised by the *exclusive formation of X-bearing sperm*. This results in all zygotes having the same chromosome constitution. Second, the chromosomes that are eliminated in the zygote are those *inherited from the father*. Third, an *imprinting process* occurs in the female, which determines that the chromosomes to be eliminated are of paternal origin. And fourth, in the cases studied, an unknown factor is produced during oogenesis that accumulates in the oocyte and then governs the elimination of the X chromosome in the developing zygote [3,4].

As mentioned above, in monogenic species, the chromosomes inherited from the father are eliminated during spermatogenesis, so that only those inherited from the mother are transmitted to the next generation. This is a process known as paternal genome elimination (PGE). The studies on PGE, found in many animals, have been mainly focused on understand the evolutionary forces that make it possible without any explicit connotation to its genetic basis [16,17,18,19,20,21,22,23,24]. From the genetic point of view, PGE is the result of two processes: an imprinting process on the paternally derived chromosomal complement during oogenesis in the mother and an unusual spermatogenesis that results in the elimination of that complement during spermatogenesis in the sons.

A general evolutionary population genetic model is presented here to analyse possible evolutionary transition pathways from the ancestral digenic XX/X0 mechanism of sex determination, based on the different initial chromosomal constitution of zygotes, to one in which all zygotes are initially identical and the chromosomal signal of sex determination is produced by the elimination of the X chromosome of paternal origin. It is assumed that new genes arise in the population, which control the aforementioned processes that characterise the monogenic mechanism, and the analysis consists of determining the putative evolutionary transition sequences of the appearance of these new genes that convert a digenic population into a monogenic one. It should be noted that in this analysis we deal exclusively with sex determination and not with dosage compensation; that is, with the process that has evolved to eliminate the differences in the two sexes between the products encoded by genes located on the sex chromosomes and those located on the autosomes. The latter process is not affected, but only the way in which the X/A chromosomal signal is produced.

## 2. Materials and Methods

### 2.1. Genetic Basis of the Model

**Figure 2:**
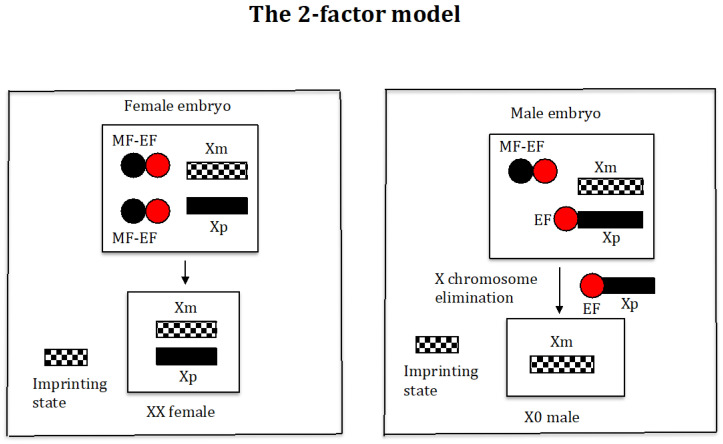
The 2-factor model. The maternal and paternal origin of the chromosomes are indicated by “m” and “p”, respectively. A maternal factor (MF) controls the elimination of the X chromosome from the soma. The gene encoding the MF is thought to be expressed only in females during oogenesis, and the MF is stored in the oocyte. The final amount of MF stored in the oocyte depends on the number of copies of the gene encoding the MF. The elimination factor (EF) binds to the X chromosome, causing its elimination; its amount is similar in all embryos. The maternal MF interacts with the EF, causing its inactivation, and its amount in embryos destined to become females is higher than in those destined to become males. The imprinted state is manifested by the inability of the maternal X chromosome to bind to the EF.

This model is based on the 2-factor model developed by Sánchez and Perondini [25] for the control of X chromosome elimination in sciarid flies (Figure 2).

Briefly, this model proposes the existence of an elimination factor (EF) (red ball) that binds to the X chromosome, causing its elimination, and its quantity is similar in all embryos. In addition, there is a maternally inherited factor (MF) (black ball) that controls X chromosome elimination by interacting with the factor (EF), causing its inactivation. The amount of the MF in embryos destined to become females is higher (two black balls) than in those destined to become males (one black ball). The imprinted state is manifested by the inability of the maternal X chromosome to bind to factor (EF).

The model analysed here explicitly considers the emergence in the population of genes, whose functions control, respectively, one of the four key processes mentioned above (see Table 1 for the biological meaning of the symbols used in this work). The gene (*s*) would be responsible for unusual spermatogenesis (AB) characterised by the segregation of maternally and paternally inherited chromosome sets from each other; that is, whilst in normal spermatogenesis the maternally and paternally derived chromosomes mix in the final sperm, in AB spermatogenesis the maternally and paternally derived chromosomes do not mix but remain separate from each other (Supplementary Material Text S1). The gene (*r*) would encode the elimination factor (EF) that binds to the X chromosome causing its elimination (Supplementary Material Text S2). The gene (*e*) would encode the maternal factor (MF) that interacts with the elimination factor (EF) causing its inactivation (Supplementary Material Text S2). The gene (*g*) would be responsible for the imprinting state of the X chromosome inherited from the mother, which prevents the binding of the elimination factor (EF) to that chromosome in the soma, and to simplify, it is considered that the same imprinting process that marks the X chromosome, to prevent its elimination in the zygote, acts on the chromosomal complement of maternal origin to prevent its elimination during spermatogenesis. The nomenclature *g*(*X0*) in males has been used to indicate that the adult comes from a mother where imprinting occurs. The new genes (*r*), (*e*), (*s*), and (*g*) are considered to be, respectively, modified genes from duplicate copies of the existing genes (*R*), (*E*), (*S*), and (*G*) whose functions have nothing to do with the process modelled here, so that the new genes acquire a new function without compromising their original functions.

The question that arises is about the dominant or recessive properties of the new genes (*s*), (*r*), and (*g*). If a single dose of the new gene is sufficient for it to exert its function, then the new gene behaves as a dominant gene. If the new gene needs to be homozygous to exert its function, then the new gene behaves as a recessive gene. This does not apply to gene (*e*), which is expressed in the mother and its product [e] accumulates in the oocyte: if the mother carries a single copy of (*e*); that is, she is heterozygous for (*e*), the amount of product [e] in the oocyte will be 1[e], whilst if the mother is homozygous for (*e*), the amount of [e] in the oocyte is 2[e]. In the first case, only one product 1[r] will be inactivated in the zygote, whereas in the second case, two products 2[r] will be inactivated.

Another question to consider is whether the new gene affects fitness—that is, the ability of an organism to reproduce and survive. Here, it is only considered positive fitness. For purposes of explanation, we consider gene (*r*). Suppose that (*r*) confers a fitness advantage to its carrier. There are two scenarios depending on whether (*r*) is dominant or recessive. If (*r*) is dominant, denoted as s^+^(*r*)dom, the fitness (*w*) of the putative genotypes are *w*(*rr*) = *w*(*Rr*) = 1 and *w*(*RR*) = 1 − µ, with (µ) being the selection coefficient. If (*r*) is recessive, denoted as s^+^(*r*)rec, the fitness (*w*) of the putative genotypes are: *w*(*rr*) = 1 and *w*(*Rr*) *= w*(*RR*) = 1 − µ, with (µ) being the selection coefficient. If gene (*r*) does not confer positive selection on its carrier, it is denoted as (*r*). The value of the selection coefficient for calculations was always 0.1. The same logic applies to the other novel genes. With respect to gene (*e*), two situations were considered regarding the stoichiometric interaction between the maternal factor [e] and the elimination factor [r]: either factor 1[e] inactivates factor 1[r] or factor 1[e*] inactivates factor 2[r]. The generational update is the adult stage.

### 2.2. Evolutionary Dynamics

An infinite panmictic population with discrete, non-overlapping generations and no migration is assumed. The reproductive factor consists of two components: mating ability (sexual selection) and the ability to produce gametes (number and types). Genes (*r*), (*e*), (*s*), and (*g*) are assumed to be mating-neutral. There is a meiotic drive effect owing to gene (*s*) in males in the presence of chromosomal imprinting that affects the type of sperm produced, since the gene (*s*) determines that only sperm carrying the maternally chromosomal inherited complement will be produced. However, it is assumed that the male produced an excess of sperm so that the male’s ability to fertilise is not compromised. Finally, for simplicity, the genes (*r*), (*e*), (*s*) and (*g*) are additionally considered autosomal and unlinked. The analysis consists of calculating the relative frequencies of all possible genotypes of males and females, respectively, across generations (Supplementary Material Text S3).

The objective of the analysis presented here is to understand the putative evolutionary transitions from a sex determination mechanism based on the chromosomal constitution of the zygote, which is fixed at fertilisation as a direct consequence of the chromosomal constitution of the gametes, to one in which the chromosomal constitution of the zygote is produced by the elimination of chromosomes of paternal origin. This manuscript contains the following analyses:

- Chromosomal imprinting is fixed in the population and occurs in the mother, and the eliminated X chromosome is of paternal origin. The 2-factor model.
- Is it possible to develop a monogenic population in the absence of chromosomal imprinting?
- Putative acquisition of chromosomal imprinting.
- A quantitative change in the interaction between the elimination factor and its maternal inhibitor modifies the genotypic formula of the monogenic state.
- Chromosomal imprinting is fixed in the population and occurs in the mother, and the eliminated X chromosome is of paternal origin. The 1-factor model.

## 3. Results

### 3.1. Chromosome Imprinting Is Fixed in the Population and Occurs in the Mother and the Eliminated X Chromosome Is of Paternal Origin. The 2-Factor Model

The study involves the appearance of genes (*e*), (*s*), and (*r*) in an XX/X0 population where gene (*g*), which causes chromosomal imprinting, is already fixed in the population; that is, (*g*) is homozygous. The resulting sequences are (*e s r*), (*e r s*), (*s e r*), (*s r e*), (*r e s*), (*r s e*) (see Table 1).

#### 3.1.1. Scenario 1A. The Gene (s) Is Dominant and the Gene (r) Is Dominant (Figure 3)

In case gene (*e*) arises under positive selection, either dominant or recessive, the population XX/X0 evolves to *XX ee SS RR gg* and *g*(*X0 ee SS RR gg*, where homozygosis for gene (*e*) makes it impossible for this population to evolve into a monogenic population. If (*e*) does not show positive selection, a new population is generated with genotypes *XX EE SS RR gg*, *XX Ee SS RR gg*, *XX ee SS RR gg*, *g*(*X0 EE SS RR gg*), *g*(*X0 Ee SS RR gg*), and *g*(*X0 ee SS RR gg*). The emergence of gene (*s*) in that new population, sequence (*e s*), whether it has a positive dominant effect or is neutral, leads to extinction owing to the lack of males. In case the gene that arises is (*r*), sequence (*e r*), this new gene is eliminated if it does not have a positive effect or the population evolves towards *XX ee SS rr gg/g*(*X0 ee SS rr gg*) in case of dominant positive selection for (*r*), where homozygosis for gene (*e*) makes it impossible for this population to evolve into a monogenic population.

**Figure 3:**
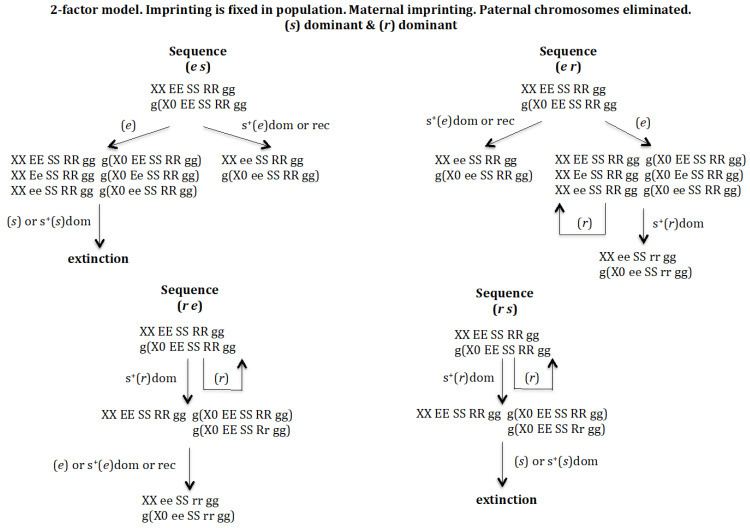
The 2-factor model. Imprinting is fixed in the population. Maternal imprinting. Paternal chromosomes eliminated. (*s*) dominant (dom) and (*r*) dominant (dom). Extinction takes place through the elimination of males.

The emergence of (*r*) without positive selection in *XX EE SS RR gg* and *g*(*X0 EE SS RR gg*) causes its elimination, whilst if (*r*) has a dominant positive effect it drives the population to a new one: *XX EE SS RR gg*, *g*(*X0 EE SS RR gg*), and *g*(*X0 EE SS Rr gg*). The arising of gene (*e*) in that population, sequence (*r e*), gives rise to the new population *XX ee SS rr gg/g*(*X0 ee SS rr gg*), which cannot evolve into a monogenic population because gene (*e*) is homozygous. If instead of gene (*e*) emerging, gene (*s*) does, sequence (*r s*), the population is driven towards extinction owing to the lack of males, regardless of whether (*s*) has a dominant positive effect or not.

It may therefore be concluded that the digenic population cannot evolve into a monogenic population under dominant conditions of genes (*s*) and (*r*).

#### 3.1.2. Scenario 1B. The Gene (s) Is Dominant and the Gene (r) Is Recessive (Figure 4)

The appearance of the gene (*r*) in the population *XX EE SS RR* gg and g(*X0 EE SS RR gg*) gives rise to a new population provided that it has a recessive positive selective value: *XX EE SS RR gg*, *XX EE SS Rr gg*, *g*(*X0 EE SS RR gg*), *g*(*X0 EE SS Rr gg*), and *g*(*X0 EE SS rr gg*). The appearance of gene (*s*) in this population leads it to the new population *XX EE ss Rr gg* and *g*(*X0 EE ss rr gg*) whether it has no positive selective value or has one (dominant). Finally, the emergence of gene (*e*) in the previous population gives rise to the monogenic population *XX Ee ss rr gg*, *XX ee ss rr gg*, *g*(*X0 EE ss rr gg*), *g*(*X0 Ee ss rr gg*), *g*(*X0 eE ss rr gg*), and *g*(*X0 ee ss rr gg*). The *XX Ee ss rr gg* are the androgenic females (they only produce males in their offspring) and the *XX ee ss rr gg* are the gynogenic females (they only produce females in their offspring).

**Figure 4:**
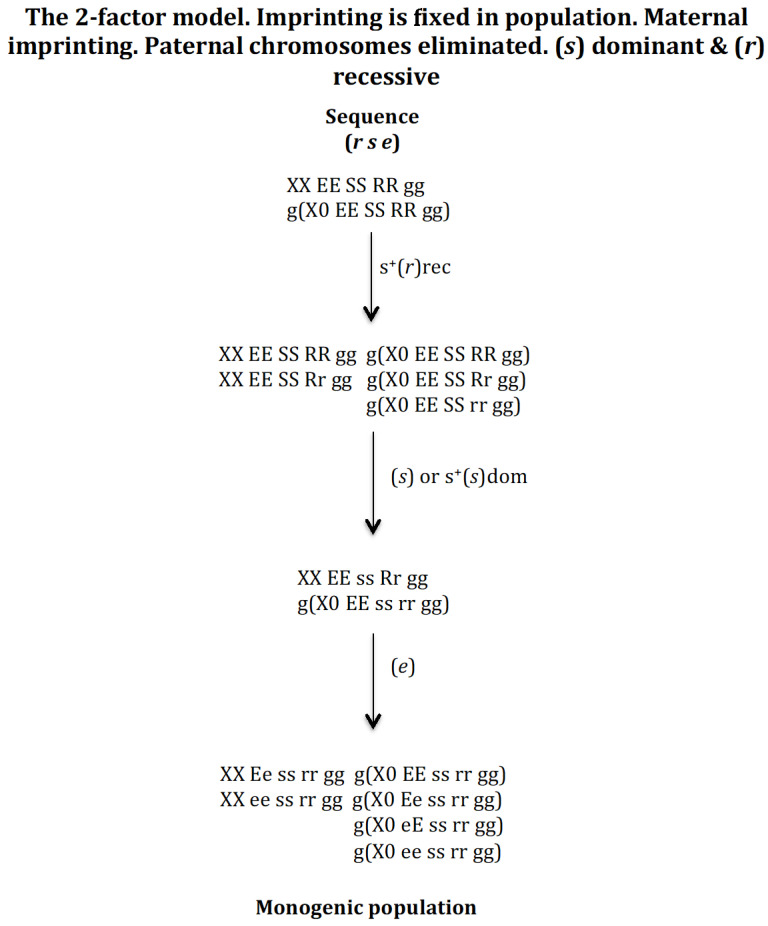
The 2-factor model. Imprinting is fixed in population. Maternal imprinting. Paternal chromosomes eliminated. (*s*) dominant (dom) and (*r*) recessive (rec). The stable states corresponding to the sequence (*r s e*) are the following. Initial state *XX EE SS RR gg* = 1, *g*(*X0 EE SS RR gg*) = 1, SR = 1. Intermediate stable state corresponding to the emergence of (*r*) with s^+^(*r*)rec *XX EE SS RR gg* = 0.519, *XX EE SS Rr gg* = 0.481, *g*(*X0 EE SS RR gg*) = 0.425, *g*(*X0 EE SS Rr gg*) = 0.393, *g*(*X0 EE SS rr gg*) = 0.182; SR = 1.22. Intermediate stable state (*r* s) corresponding to the emergence of (*s*) in the previous population with no positive selection for (*s*) *XX EE ss Rr gg* = 1, *g*(*X0 EE ss rr gg*) = 1, SR = 1.11; or with dominant positive selection for s^+^(*s*)dom *XX EE ss Rr gg* = 1, *g*(*X0 EE ss rr gg*) = 1, SR = 1. Final state corresponding to the evolutionary sequence (*r s e*) after emergence of gene (*e*) in any of the two previous (*r s*) stable states: *XX Ee ss rr gg* = 0.5; *XX ee ss rr gg* = 0.5; *g*(*X0 EE ss rr gg*) = 0.25; *g*(*X0 Ee ss rr gg*) = 0.25; *g*(*X0 eE ss rr gg*) = 0.25; *g*(*X0 ee ss rr gg*) = 0.25; SR = 1.

It may therefore be concluded that the digenic population can evolve into a monogenic population through the sequence (*r s e*) under dominant condition of gene (*s*) and recessive condition of gene (*r*).

#### 3.1.3. Scenario 1C. The Gene (s) Is Recessive and the Gene (r) Is Dominant (Figure 5)

**Figure 5:**
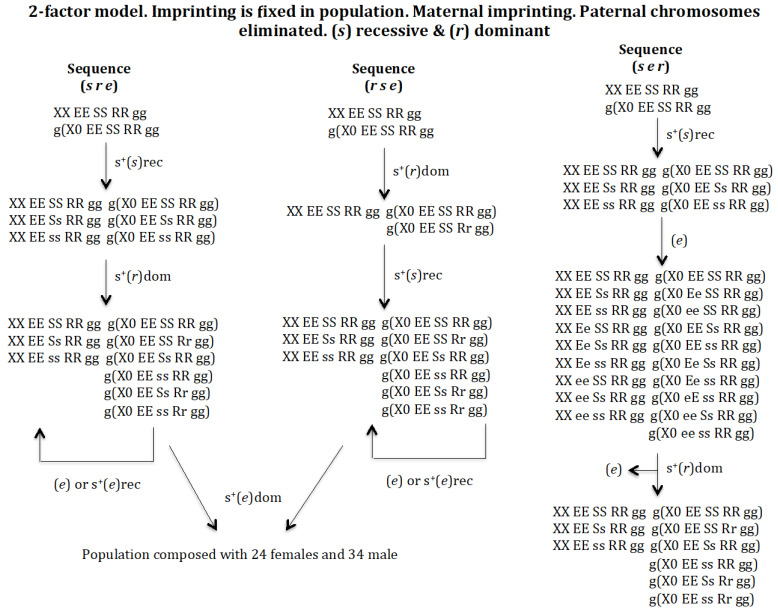
The 2-factor model. Imprinting is fixed in population. Maternal imprinting. Paternal chromosomes eliminated. (*s*) recessive (rec) and (*r*) dominant (dom). The population of the final state corresponding to the evolutionary sequence (*s e r*) is: *XX EE SS RR gg* = 0.239, *XX EE Ss RR gg* = 0.493, *XX EE ss RR gg* = 0.267, *g*(*X0 EE SS RR gg*) = 0.304, *g*(*X0 EE SS Rr gg*) = 0.033, *g*(*X0 EE Ss RR gg*) = 0.451, *g*(*X0 EE ss RR gg*) = 0.144, *g*(*X0 EE Ss Rr gg*) = 0.050 and *g*(*X0 EE ss Rr gg*) = 0.016. SR = 0.78.

**Sequence (*s r e*)**. The appearance of a gene (*s*) in the population *XX EE SS RR* gg and g(*X0 EE SS RR gg*) gives rise to a new population provided it has a recessive positive selective value: *XX EE SS RR gg*, *XX EE Ss RR gg*, *XX EE ss RR gg*, *g*(*X0 EE SS RR gg*), *g*(*X0 EE Ss RR gg*), and *g*(*X0 EE ss RR gg*). The emergence of gene (*r*) in the previous population leads to another population where genes (*s*) and (*r*) coexist: *XX EE SS RR gg*, *XX EE Ss RR gg*, *XX EE ss RR gg*, *g*(*X0 EE SS RR gg*), *g*(*X0 EE SS Rr gg*), *g*(*X0 EE Ss RR gg*), *g*(*X0 EE ss RR gg*), *g*(*X0 EE Ss Rr gg*), and *g*(*X0 EE ss Rr gg*). This transition is possible if the new gene (*r*) has a dominant positive selection value, otherwise gene (*r*) is eliminated (result not represented in Figure 5). Finally, the emergence of gene (*e*) in the previous population evolves into a population composed of 24 female genotypes and 34 male genotypes (results not represented in Figure 5).

**Sequence (*r s e*)**. The appearance of gene (*r*) in population *XX EE SS RR* gg and g(*X0 EE SS RR gg*) gives rise to a new population provided that it has a positive selective value (dominant): *XX EE SS RR gg*, *g*(*X0 EE SS RR gg*, *and g*(*X0 EE SS Rr gg*). The appearance of gene (*s*) with recessive positive selection in the previous population gives rise to another population where genes (*s*) and (*r*) coexist: *XX EE SS RR gg*, *XX EE Ss RR gg*, *XX EE ss RR gg*, *g*(*X0 EE SS RR gg*), *g*(*X0 EE SS Rr gg*), *g*(*X0 EE Ss RR gg*), *g*(*X0 EE ss RR gg*), *g*(*X0 EE ss RR gg*), *g*(*X0 EE Ss Rr gg*), and *g*(*X0 EE ss Rr gg*). Finally, the emergence of gene (*e*) in the previous population evolves into a population composed of 24 female genotypes and 34 male genotypes (results not represented in Figure 5).

**Sequence (*s e r*)**. The appearance of gene (*s*) in population *XX EE SS RR* gg and g(*X0 EE SS RR gg*) gives rise to a new population, provided that it has a recessive positive selective value: *XX EE SS RR gg*, *XX EE Ss RR gg*, *XX EE ss RR gg*, *g*(*X0 EE SS RR gg*), *g*(*X0 EE Ss RR gg*), and *g*(*X0 EE ss RR gg*). The appearance of gene (*e*) in that population without a positive selection value gives rise to a new population *XX EE SS RR gg*, *XX EE Ss RR gg*, *XX EE ss RR gg*, *XX Ee SS RR gg*, *XX Ee Ss RR gg*, *XX Ee ss RR gg*, *XX ee SS RR gg*, *XX ee Ss RR gg*, *XX ee ss RR gg*, *g*(*X0 EE SS RR gg*), *g*(*X0 Ee SS RR gg*), *g*(*X0 ee SS RR gg*), *g*(*X0 EE Ss RR gg*), *g*(*X0 EE ss RR gg*), *g*(*X0 Ee Ss RR gg*), *g*(*X0 Ee ss RR gg*), *g*(*X0 eE ss RR gg*), *g*(*X0 ee Ss RR gg*), and *g*(*X0 ee ss RR gg*). Gene (*e*) is eliminated if it had a recessive positive selection value, or in the case of a dominant positive selection value it generates a new population homozygous for gene (*e*), which makes it impossible for this population to evolve into a monogenic population (results not represented in Figure 5). The appearance of the gene (*r*), with dominant positive selective value, in the previous population causes the generation of a new population *XX EE SS RR gg*, *XX EE Ss RR gg*, *XX EE ss RR gg*, *g*(*X0 EE SS RR gg*), *g*(*X0 EE SS Rr gg*), *g*(*X0 EE Ss RR gg*), *g*(*X0 EE ss RR gg*), *g*(*X0 EE Ss Rr gg*), and *g*(*X0 EE ss Rr gg*) after the elimination of gene (*e*).

It may therefore be concluded that the digenic population cannot evolve into a monogenic population under recessive conditions of gene (*s*) and dominance of gene (*r*).

#### 3.1.4. Scenario 1D. The Gene (s) Is Recessive and the Gene (r) Is Recessive (Figure 6)

**Figure 6:**
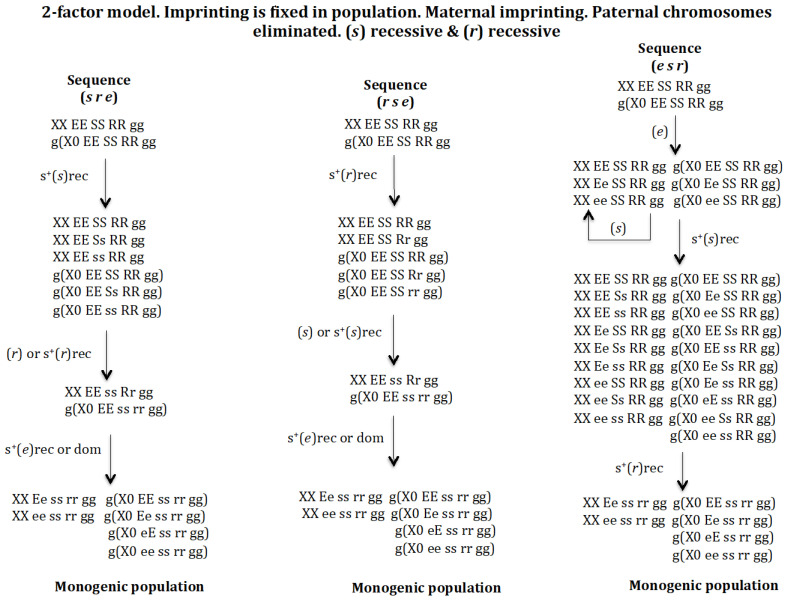
The 2–factor model. Imprinting is fixed in population. Maternal imprinting. Paternal chromosomes eliminated. (*s*) recessive (rec) and (*r*) recessive (rec). The stable states corresponding to the sequence (*s r e*) are the following. Initial state *XX EE SS RR gg* = 1, *g*(*X0 EE SS RR gg*) = 1, SR = 1. Intermediate stable state corresponding to the emergence of (*s*) with s^+^(*s*)rec *XX EE SS RR gg* = 0.157, *XX EE Ss RR gg* = 0.464, *XX EE ss RR gg* = 0.378, g(X0 EE SS RR gg) = 0.252, g(X0 EE S RR gg) = 0.523, *g*(*X0 EE ss RR gg*) = 0.223, SR = 0.62. Intermediate stable state (*s r*) corresponding to the emergence of (*r*) in the previous population with or without positive selection for s^+^(*r*)rec *XX EE ss Rr gg* = 1, *g*(*X0 EE ss rr gg*) = 1, SR = 1. Final state corresponding to the evolutionary sequence (*s r e*) corresponding to the emergence of (*e*) in the previous population with no positive selection for (*e*) *XX Ee ss rr gg* = 0.5, *XX ee ss rr gg* = 0.5, *g*(*X0 EE ss rr gg*) = 0.25, *g*(*X0 Ee ss rr gg*) = 0.25, *g*(*X0 eE ss rr gg*) = 0.25, *g*(*X0 ee ss rr gg*) = 0.25, SR = 1; or with recessive positive selection for s^+^(*e*)rec *XX Ee ss rr gg* = 0.487, *XX ee ss rr gg* = 0.512, *g*(*X0 EE ss rr gg*) = 0.224, *g*(*X0 Ee ss rr gg*) = 0.262, *g*(*X0 eE ss rr gg*) = 0.249, *g*(*X0 ee ss rr gg*) = 0.262, SR = 0.93; or with dominant positive selection for s^+^(*e*)dom *XX Ee ss rr gg* = 0.459, *XX ee ss rr gg* = 0.540, *g*(*X0 EE ss rr gg*) = 0.236, *g*(*X0 Ee ss rr gg*) = 0.249, *g*(*X0 eE ss rr gg*) = 0.236, *g*(*X0 ee ss rr gg*) = 0.277, SR = 0.83. The stable states corresponding to the sequence (*r s e*) are the following. Initial state *XX EE SS RR gg* = 1, *g*(*X0 EE SS RR gg*) = 1, SR = 1. Intermediate stable state corresponding to the emergence of (*r*) with s^+^(*r*)rec *XX EE SS RR gg* = 0.519, *XX EE Ss RR gg* = 0.480, *g*(*X0 EE SS RR gg*) = 0.425, *g*(*X0 EE SS Rr gg*) = 0.393, *g*(*X0 EE SS rr gg*) = 0.182, SR = 1.2. Intermediate stable state (*r s*) corresponding to the emergence of (*s*) in the previous population without positive selection for (*s*) *XX EE ss Rr gg* = 1, *g*(*X0 EE ss rr gg*) = 1, SR = 1.11; or with positive selection for s^+^(*s*) = 0.1rec *XX EE ss Rr gg* = 1, *g*(*X0 EE ss rr gg*) = 1, SR = 1.05. Final state corresponding to the evolutionary sequence (*s r e*) corresponding to the emergence of (*e*) in the previous population with no selection or recessive positive selection s^+^(*s*)rec *XX Ee ss rr gg* = 0.5, *XX ee ss rr gg* = 0.5, *g*(*X0 EE ss rr gg*) = 0.25, *g*(*X0 Ee ss rr gg*) = 0.25, *g*(*X0 eE ss rr gg*) = 0.25, *g*(*X0 ee ss rr gg*) = 0.25, SR = 1; or with dominant positive selection s^+^(*s*)dom *XX Ee ss rr gg* = 0.494, *XX ee ss rr gg* = 0.506, *g*(*X0 EE ss rr gg*) = 0.237, *g*(*X0 Ee ss rr gg*) = 0.256, *g*(*X0 eE ss rr gg*) = 0.259, *g*(*X0 ee ss rr gg*) = 0.256, SR = 0.96. The stable states corresponding to the sequence (*e s r*) are the following. Initial state *XX EE SS RR gg* = 1, *g*(*X0 EE SS RR gg*) = 1, SR = 1. Intermediate stable state corresponding to the emergence of (*e*) without positive selection is *XX EE SS RR gg* = 0.902, *XX Ee SS RR gg* = 0.095, *XX ee SS RR gg* = 0.0025, *g*(*X0 EE SS RR gg*) = 0.902, *g*(*X0 Ee SS RR gg*) = 0.095, *g*(*X0 ee SS RR gg*) = 0.0025, SR = 1. Intermediate stable state (*e s*) corresponding to the emergence of (*s*) in the previous population with recessive positive selection for s^+^(*s*)rec *XX EE SS RR gg* = 0.147, *XX EE Ss RR gg* = 0.423, *XX EE ss RR gg* = 0.345, *XX Ee SS RR gg* = 0.013, *XX Ee Ss RR gg* = 0.034, *XX Ee ss RR gg* = 0.033, *XX ee SS RR gg* = 0.0003, *XX ee Ss RR gg* = 0.0009, *XX ee ss RR gg* = 0.0007, *g*(*X0 EE SS RR gg*) = 0.230, *g*(*X0 Ee SS RR gg*) = 0.022, *g*(*X0 ee SS RR gg*) = 0.0005, *g*(*X0 EE Ss RR gg*) = 0.478, *g*(*X0 EE ss RR gg*) = 0.204, *g*(*X0 Ee Ss RR gg*) = 0.045, *g*(*X0 Ee ss RR gg*) = 0.009, *g*(*X0 eE ss RR gg*) = 0.009, *g*(*X0 ee Ss RR gg*) = 0.0011, *g*(*X0 ee ss RR gg*) = 0.0004, SR = 0.62. Final state corresponding to the evolutionary sequence (*e s r*) corresponding to the emergence of (*r*) in the previous population with recessive positive selection for s^+^(*r*) *XX Ee ss rr gg* = 0.5, *XX ee ss rr gg* = 0.5, *g*(*X0 EE ss rr gg*) = 0.25, *g*(*X0 Ee ss rr gg*) = 0.25, *g*(*X0 eE ss rr gg*) = 0.25, *g*(*X0 ee ss rr gg*) = 0.25, SR = 1.

**Sequence (*s r e*)**. The appearance of gene (s) in population *XX EE SS RR gg* and *g*(*X0 EE SS RR gg*) gives rise to a new population provided it has a positive recessive selective value: *XX EE SS RR gg*, *XX EE Ss RR gg*, *XX EE ss RR gg*, *g*(*X0 EE SS RR gg*), *g*(*X0 EE Ss RR gg*), and *g*(*X0 EE ss RR gg*). The appearance of gene (*r*) in the previous population with or without a recessive positive selection value gives rise to another population where genes (*s*) and (*r*) coexist: *XX EE ss Rr gg* and *g*(*X0 EE ss rr gg*). Finally, the appearance of gene (*e*) in the previous population evolves to the monogenic population *XX Ee ss rr gg*, *XX ee ss rr gg*, *g*(*X0 EE ss rr gg*), *g*(*X0 Ee ss rr gg*), *g*(*X0 eE ss rr gg*), and *g*(*X0 ee ss rr gg*) regardless of whether gene (*e*) has a recessive or dominant positive selection value. If gene (*e*) does not have a positive selective value, a non-monogenic population is generated where digenic and monogenic females coexist (result not represented in Figure 6).

**Sequence (*r s e*)**. The emergence of gene (*r*) in population *XX EE SS RR gg* and *g*(*X0 EE SS RR gg*) gives rise to a new population provided that it has a recessive positive selective value: *XX EE SS RR gg*, *XX EE SS Rr gg*, *g*(*X0 EE SS RR gg*), *g*(*X0 EE SS Rr gg*), and *g*(*X0 EE SS rr gg*). The appearance of gene (*s*) in the previous population gives rise to another population where genes (*s*) and (*r*) coexist: *XX EE ss Rr gg* and *g*(*X0 EE ss rr gg*), if (*s*) does not have or has a recessive positive selection value. Finally, the emergence of gene (*e*) in the previous population evolves into the monogenic population *XX Ee ss rr gg*, *XX ee ss rr gg*, *g*(*X0 EE ss rr gg*), *g* (*X0 Ee ss rr gg*), *g*(*X0 eE ss rr gg*), and *g*(*X0 ee ss rr gg*), regardless of whether gene (*e*) has a recessive or dominant positive selection value. If gene (*e*) does not have a positive selective value, a non-monogenic population is generated where digenic and monogenic females coexist (result not represented in Figure 6).

**Sequence (*e s r*)**. The appearance of gene (*e*) in population *XX EE SS RR gg* and *g*(*X0 EE SS RR gg*) gives rise to a new population provided that it does not have a positive recessive or dominant selective value: *XX EE SS RR gg*, *XX Ee SS RR gg*, *XX ee SS RR gg*, *g*(*X0 EE SS RR gg*), *g*(*X0 Ee SS RR gg*), and *g*(*X0 ee SS RR gg*). The emergence of gene (*s*), with positive recessive selection value, drives the population towards a new one composed of 9 female genotypes and 10 male genotypes where genes (*s*) and (*r*) coexist (see sequence (*e s r*)) in Figure 6). If gene (s) does not have a positive selection value, it is eliminated. Finally, the appearance of gene (*r*) in the previous population evolves towards the monogenic population *XX Ee ss rr gg*, *XX ee ss rr gg*, *g*(*X0 EE ss rr gg*), *g* (*X0 Ee ss rr gg*), *g*(*X0 eE ss rr gg*), and *g*(*X0 ee ss rr gg*), provided that gene (*r*) has a recessive positive selection value. If (*r*) does not have a positive selection value, a non-monogenic population is formed where digenic and monogenic females coexist (result not represented in Figure 6).

It may therefore be concluded that the digenic population can evolve into a monogenic population through the sequences (*s r e*), (*r s e*) and (*e s r*) under recessive conditions of gene (*s*) and gene (*r*).

### 3.2. Is It Possible to Develop a Monogenic Population in the Absence of Chromosomal Imprinting? (Figure 7)

**Figure 7:**
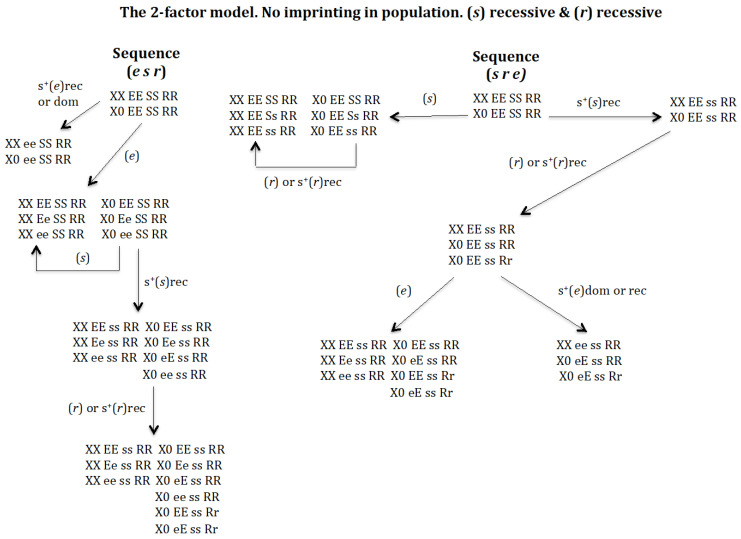
The 2-factor model. Non-imprinting. (*s*) recessive (rec) and (*r*) recessive (rec). The stable states corresponding to the sequence (*e s r*) are the following. Initial state *XX EE SS RR* = 1, *X0 EE SS RR* = 1, SR = 1. Intermediate stable state corresponding to the emergence of (*e*) without positive selection for (*e*) *XX EE SS RR* = 0.9025, *XX Ee SS RR* = 0.095, *XX ee SS RR* = 0.0025, *X0 EE SS RR* = 0.9025, *X0 Ee SS RR* = 0.095, X0 *ee SS RR* = 0.0025, SR = 1. Intermediate stable state (*e s*) corresponding to the emergence of (*s*) in the previous population with s^+^(*s*)rec *XX EE ss RR* = 0.912, *XX Ee ss RR* = 0.086, *XX ee ss RR* = 0.002, *X0 EE ss RR* = 0.912, *X0 Ee ss RR* = 0.043, *X0 eE ss RR* = 0.043, *X0 ee ss RR* = 0.002, SR = 1. Final state corresponding to the evolutionary sequence (*e s r*) after emergence of gene (*r*) in the previous population *XX EE ss RR* = 0.92, *XX Ee ss RR* = 0.078, *XX ee ss RR* = 0.002, *X0 EE ss RR* = 0.764, *X0 Ee ss RR* = 0.039, *X0 eE ss RR* = 0.032, *X0 ee ss RR* = 0.002, *X0 EE ss Rr* = 0.156, *X0 eE ss Rr* = 0.007, SR = 1 regardless of whether or not there is recessive positive selection for gene (*r*). The stable states corresponding to the sequence (*s r e*) are the following. Initial state *XX EE SS RR* = 1, *X0 EE SS RR* = 1, SR = 1. Intermediate stable state corresponding to the emergence of (*s*) with s^+^(*s*)rec *XX EE ss RR* = 1, *X0 EE ss RR* = 1, SR = 1. Intermediate stable state (*s r*) corresponding to the emergence of (*r*) in the previous population *XX EE ss RR* = 1, *X0 EE ss RR* = 0.833, *X0 EE ss Rr* = 0.167, SR = 1, regardless of whether or not there is recessive positive selection for gene (*r*). Final states corresponding to the evolutionary sequence (*s r e*) after emergence of gene (*e*) without positive selection in the previous population *XX EE ss RR* = 0.871, *XX Ee ss RR* = 0.125, *XX ee ss RR* = 0.004, *X0 EE ss RR* = 0.793, *X0 eE ss RR* = 0.057, *X0 EE ss Rr* = 0.14, *X0 eE ss Rr* = 0.01, SR = 1; or *XX ee ss RR* = 1, *X0 eE ss RR* = 0.85, *X0 eE ss Rr* = 0.15, SR = 1, regardless of whether there is dominance or recessive positive selection for gene (*e*).

To address this question, the scenario that allows the highest potential for evolutionary sequences under recessive conditions for (*s*) and (*r*) was analysed. Among the six putative (*e s r*), (*e r s*), (*s e r*), (*s r e*), (*r e s*), (*r s e*) evolutionary transitions that are possible, only the sequences (*e s r*) and (*s r e*) occur but does not give rise to a monogenic population (Figure 7). The sequence (*r s e*) is not feasible because if the gene (*r*) emerges in population *XX EE SS RR* and *X0 EE SS RR* it is eliminated even under conditions of recessive positive selection for (*r*).

**Sequence (*e s r*)**. The appearance of gene (*e*) in the population *XX EE SS RR* and *X0 EE SS RR* leads to the new population *XX ee SS RR*, *X0 ee SS RR* if (*e*) has a dominant or recessive positive selective value. This population cannot evolve into a monogenic population because gene (*e*) is homozygous (Figure 7). In the absence of positive selection for (*e*), the new population is *XX EE SS RR*, *XX Ee SS RR*, *XX ee SS RR*, *X0 EE SS RR*, *X0 Ee SS RR* and *X0 ee SS RR gg* (Figure 7). The appearance of (*s*) in this population gives rise to the population *XX EE ss RR*, *XX Ee ss RR*, *XX ee ss RR*, *X0 EE ss RR*, *X0 Ee ss RR*, *X0 eE ss R*R and *X0 ee ss RR*, provided that (*s*) has a recessive positive selective value; otherwise, the emerging gene (*s*) is eliminated (Figure 7). If instead of (*s*), it is (*r*) the emerging gene, (*r*) is eliminated whether or not it has a recessive positive selective value (results not represented in Figure 7). Finally, the appearance of gene (*r*) in the previous population corresponding to the sequence (*e s*) leads to the new population *XX EE ss RR*, *XX Ee ss RR*, *XX ee ss RR*, *X0 EE ss RR*, *X0 Ee ss RR*, *X0 eE ss RR*, *X0 ee ss RR*, *X0 EE ss Rr* and *X0 eE ss Rr*, whether or not (*r*) has a recessive positive selective value. This new population is not monogenic owing to the existence of the digenic female *XX EE ss RR* (Figure 7).

**Sequence (*s r e*)**. The emergence of gene (*s*) in population *XX EE SS RR* and *X0 EE SS RR* gives rise to the new population *XX EE SS RR*, *XX EE Ss RR*, *XX EE ss RR*, *X0 EE SS RR*, *X0 EE Ss RR*, and *X0 EE ss RR* in the absence of positive recessive selection for (*s*). The arising of (*r*) in that population, with or without positive selection for (*r*), causes the deletion of (*r*). The emergence of gene (*s*) in the population *XX EE SS RR* and *X0 EE SS RR* leads to the new population *XX EE ss RR* and *X0 EE ss RR*, when (*s*) shows a recessive positive selective value. The emergence of (*r*) in that population causes its evolution into *XX EE ss RR*, *X0 EE ss RR*, and *X0 EE ss Rr*, whether or not (*r*) has a recessive positive selective value (Figure 7). The later emergence of (*e*) in that population, sequence (*s r e*), gives rise to two non-monogenic populations. One population is *XX EE ss RR*, *XX Ee ss RR*, *XX ee ss RR*, *X0 EE ss RR*, *X0 eE ss RR*, *X0 EE ss Rr*, and *X0 eE ss Rr* under conditions of absence of positive selection for (*e*). This population is not monogenic owing to the presence of digenic female *XX EE ss RR*. The other non-monogenic population is *XX ee ss RR*, *X0 eE ss RR* and *X0 eE ss Rr*, generated under conditions of positive selection for (*e*), either dominant or recessive (see Figure 7). This population is not monogenic because contains only one female genotype homozygous for gene (*e*).

It may therefore be concluded that the process of genomic imprinting is a necessary condition for the generation of a monogenic population.

### 3.3. Putative Acquisition of Chromosomal Imprinting (Figure 8)

**Figure 8:**
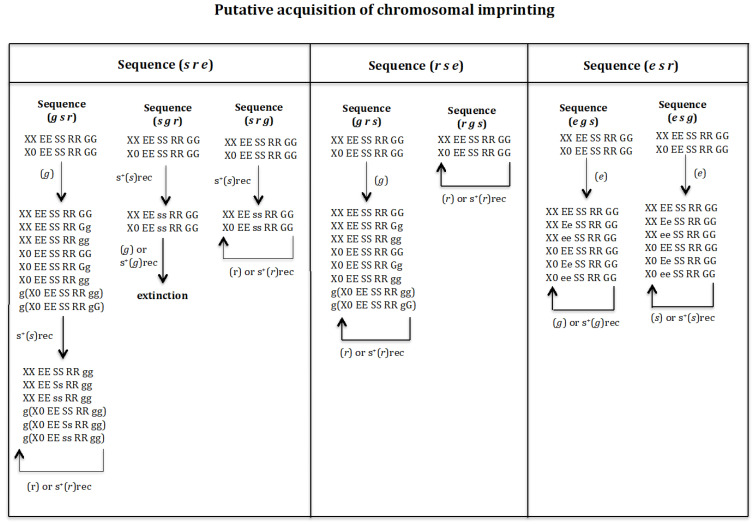
Putative acquisition of imprinting. It should be noted that the initial population lacks imprinting, and the order of appearance of gene (*g*) with respect to genes (*s*), (*r*) and (*e*) is analysed to determine if the monogenic state is reached. The sequences (*g s r*), (*s g r*), and (*s r g*) in Figure 8 correspond to the evolutionary sequence (*s r e*) that results in the formation of a monogenic population under fixation conditions for imprinting; that is homozygous for gene (*g*) (Figure 6). The sequence (*g s r*) in Figure 8 corresponds to the assumption that gene (*g*) is the first to appear, followed by gene (*s*), and finally gene (*r*). The sequence (*s g r*) in Figure 8 corresponds to the assumption that gene (*s*) is the first to appear, followed by gene (*g*) and finally gene (*r*). The sequence (*s r g*) in Figure 8 corresponds to the assumption that gene (*s*) is the first to appear, followed by gene (*r*) and finally gene (*g*). The sequences (*g r s*) and (*r g s*) in Figure 8 correspond to the evolutionary sequence (*r s e*) that results in the formation of a monogenic population under fixation conditions for imprinting; that is homozygous for gene (*g*) (Figure 6). The sequence (*g r s*) in Figure 8 corresponds to the assumption that gene (*g*) is the first to appear, followed by gene (*r*), and finally gene (*s*). The sequence (*r g s*) in Figure 8 corresponds to the assumption that gene (*r*) is the first to appear, followed by gene (*g*), and finally gene (*s*). The sequences (*e g s*) and (*e s g*) in Figure 8 correspond to the evolutionary sequence (*e s r*) that results in the formation of a monogenic population under fixation conditions for imprinting; that is homozygous for gene (*g*) (Figure 6). The sequence (*e g s*) in Figure 8 corresponds to the assumption that gene (*e*) is the first to appear, followed by gene (*g*), and finally gene (*s*). The sequence (*e s g*) in Figure 8 corresponds to the assumption that gene (*e*) is the first to appear, followed by gene (*s*), and finally gene (*g*).

It was previously shown that, when imprinting is considered to be already present (fixed) in the population, the sequences (*s r e*), (*r s e*), and (*e s r*) transform a digenic population into a monogenic one. Next, we will analyse if those sequences can generate a monogenic population if gene (*g*) was not present in the initial digenic population but it arises at the beginning or at an intermediate state or at the end state towards the putative monogenic state. For sequence (*s r e*), the following transitions were analysed: (*g s r*), (*s g r*), and (*s r g*). For sequence (*r s e*), the following transitions were analysed: (*g r s*) and (*r g s*). And for sequence (*e s r*), the following transitions were analysed (*e g s*) and (*e s g*). See Figure 8.

**Sequence (*g s r*).** Gene (*g*) is considered to arise in population *XX EE SS RR GG* and *X0 EE SS RR GG*. If it has a recessive or dominant positive selection effect, the population evolves to *XX EE SS RR gg* and *g*(*X0 EE SS RR gg*) and then we are in the situation analysed above (Figure 6). If (*g*) does not have a positive selection effect, the initial population evolves to population *XX EE SS RR GG*, *XX EE SS RR Gg*, *XX EE SS RR gg*, *X0 EE SS RR GG*, *X0 EE SS RR Gg*, *X0 EE SS RR gg*, *g*(*X0 EE SS RR gg*) and *g*(*X0 EE SS RR gG*). The appearance of (*s*) in that population with a recessive positive effect gives rise to a new population *XX EE SS RR gg*, *XX EE Ss RR gg*, *XX EE ss RR gg*, *g*(*X0 EE SS RR gg*), *g*(*X0 EE Ss RR gg*), *g*(*X0 EE ss RR gg*). If in this population, the gene (*r*) arises with or without recessive positive selection effect, it is eliminated and the monogenic state is not reached.

**Sequence (*s g r*).** Gene (*s*) is considered to have first arisen in population *XX EE SS RR GG* and *X0 EE SS RR GG* with a recessive positive effect, giving rise to population *XX EE ss RR GG* and *X0 EE ss RR GG*. The appearance of gene (*g*) in that population, with or without recessive positive selection, causes its extinction owing to a lack of males.

**Sequence (*s r g*).** Gene (*s*) is considered to have first arisen in population *XX EE SS RR GG* and *X0 EE SS RR GG* with a recessive positive effect, giving rise to population *XX EE ss RR GG* and *X0 EE ss RR GG*. The appearance of gene (*r*) in that population, with or without recessive positive selection, causes its elimination.

**Sequence (*g r s*).** Gene (*g*) is considered to have initially arising in population *XX EE SS RR GG* and *X0 EE SS RR GG*. If it has a recessive or dominant positive selection effect, the population evolves to *XX EE SS RR gg* and *g*(*X0 EE SS RR gg*) and then we are in the situation analysed above (Figure 6). If (*g*) does not have a positive selection effect, the initial population evolves to population *XX EE SS RR GG*, *XX EE SS RR Gg*, *XX EE SS RR gg*, *X0 EE SS RR GG*, *X0 EE SS RR Gg*, *X0 EE SS RR gg*, *g*(*X0 EE SS RR gg*) and *g*(*X0 EE SS RR gG*). The emergence of (*r*) in that population, with or without a recessive positive effect, causes its elimination.

**Sequence (*r g s*).** Gene (*r*) is considered to have first arisen in population *XX EE SS RR GG*, *X0 EE SS RR GG*, with or without recessive positive selection effect, being eliminated.

**Sequence (*e g s*).** Gene (*e*) is considered to have first arisen in population *XX EE SS RR GG*, *X0 EE SS RR GG*. If gene (*e*) had a recessive or dominant positive selection value, a new population is generated *XX ee SS gg* and *g*(*X0 ee SS gg*), where homozygosis for gene (*e*) prevents the formation of a monogenic population (result not shown in Figure 8). If gene (*e*) does not have a positive selection value, the population evolves into a new one: *XX EE SS RR GG*, *XX Ee SS RR GG*, *XX ee SS RR GG*, *X0 EE SS RR GG*, *X0 Ee SS RR GG*, *X0 ee SS RR GG*. If gene (*g*) arises in that population, it is eliminated, whether it has no positive selection value or is recessive. If gene (*g*) had a dominant positive selective value, the population evolves to the new one *XX ee SS gg* and *g*(*X0 ee SS gg*), (result not shown in Figure 8), where homozygosis for (*e*) prevents that the population could evolve into a monogenic population.

**Sequence (*e s g*).** Gene (*e*) is considered to have first arisen in population *XX EE SS RR GG*, *X0 EE SS RR GG*. If gene (*e*) had a recessive or dominant positive selection value, a new population is generated *XX ee SS gg* and *g*(*X0 ee SS gg*), where homozygosis for gene (*e*) prevents the formation of a monogenic population (result not shown in Figure 8). If gene (*e*) does not have a positive selective value, the population evolves into a new one: *XX EE SS RR GG*, *XX Ee SS RR GG*, *XX ee SS RR GG*, *X0 EE SS RR GG*, *X0 Ee SS RR GG*, *X0 ee SS RR GG*. If gene (*s*) arises in that population, it is eliminated, whether it has no positive selection value or is recessive. The result is that the population cannot evolve into a monogenic population.

It may therefore be concluded that the gene (*g*) must be homozygous in the initial digenic population *XX EE SS RR gg* and *g*(*X0 EE SS RR gg*) for it to evolve into a monogenic population following the posterior appearance of genes (*e*), (*s*) and (*r*) (see later in Discussion).

### 3.4. A Quantitative Change in the Interaction Between the Elimination Factor and Its Maternal Inhibitor Modifies the Genotypic Formula of the Monogenic State (Figure 9)

The analysis described above is based on the assumption that the stoichiometry of the interaction between the elimination factor [r] and its maternal inhibitor [e] follows the rule 1[e]:1[r]. Could a change in the stoichiometry of this interaction, which is now 1[e*]:2[r], lead to a change in the final genotypic formula of the monogenic population?

**Figure 9:**
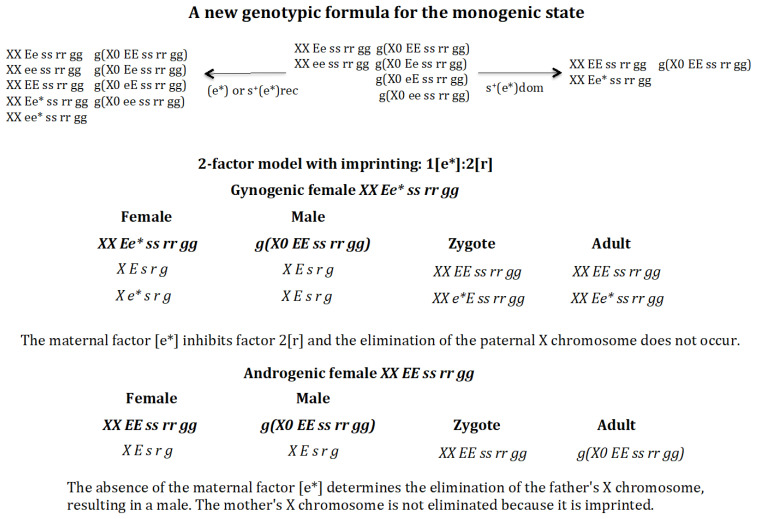
A new genotypic formula for the monogenic state. A quantitative change in the interaction between the elimination factor and its maternal inhibitor from 1[e]:1[r] to 1[e*]:2[r] modifies the genotypic formula of the monogenic state. The final state population corresponding to the appearance of gene (e*) in the absence of positive selection or recessive positive selection for (e*) is as follows: *XX Ee ss rr gg* = 0.499, *XX ee ss rr gg* = 0.498, *XX EE ss rr gg* = 0.0004, XX Ee* ss rr gg = 0.001, XX ee* ss rr gg = 0.001, *g*(*X0 EE ss rr gg*) = 0.250, *g*(*X0 Ee ss rr gg*) = 0.249, *g*(*X0 eE ss rr gg*) = 0.249, *g*(*X0 ee ss rr gg*) = 0.249, SR = 0.99. The final state population corresponding to the appearance of gene (e*) with dominant positive selection is as follows: *XX EE ss rr gg* = 0.474, XX Ee* ss rr gg = 0.526, *g*(*X0 EE ss rr gg*) = 1, SR = 1.

The monogenic state is composed of the following genotypes: *XX Ee ss rr gg* (androgenic female), *XX ee ss rr gg* (gynogenic female), *g*(*X0 EE ss rr gg*), *g*(*X0 Ee ss rr gg*), *g*(*X0 eE ss rr gg*), and *g*(*X0 ee ss rr gg*). Suppose that gene (*e*) mutates to allele (e*), characterised by a higher inhibitory efficiency against the elimination factor [r], with the stoichiometry of the interaction becoming 1[e*]:2[r]. In this case, there is a change in the genotypic formula for the monogenic state (Figure 9). In the absence of positive selection for (e*) or with recessive positive selection, the monogenic population loses its monogenic identity, since digenic females *EE ss rr gg* exist. However, if the new allele (e*) has a dominant positive selection effect, the population remains monogenic, but its genotypic composition changes to one composed of gynogenic (XX Ee* ss rr gg) and androgenic (*XX EE ss rr gg*) females and a single male genotype *g*(*X0 EE ss rr gg*). The genotype formula is explained in Figure 9.

The gynogenic female XX Ee* ss rr gg produces oocytes *X E s r g* and X e* s r g, which carry factor 1[e*]. The males *g*(*X0 EE ss rr gg*) produce sperm *X E s r g*. Two zygotes *XX EE ss rr gg* and XX Ee* ss rr gg are formed. In both cases, factor 1[e*] inhibits the elimination factor 2[r], giving rise to the adults females *XX EE ss rr gg* (androgenic) and XX Ee* ss rr gg (gynogenic).

The androgenic female *XX EE ss rr gg* produces oocytes *X E s r g*, which do not carry factor 1[e*]. Males *g*(*X0 EE ss rr gg*) produce the sperm *X E s r g*. The zygote *XX EE ss rr gg* is generated. The elimination factor 2[r] eliminates the X chromosome from the father, but not the X chromosome from the mother, since the latter is imprinted and protected from interaction with the elimination factor [r]. The result is the generation of males *g*(*X0 EE ss rr gg*).

### 3.5. Chromosome Imprinting Is Fixed in the Population and Occurs in the Mother and the Eliminated X Chromosome Is of Paternal Origin. The 1-Factor Model (Figure 10)

The model presented above is a 2-factor model, since the number of chromosomes eliminated in the zygote is the result of the interaction between two factors [r] and [e]. Factor [r] interacts with the X chromosome, causing its elimination; factor [e] interacts with factor [r] causing its inhibition, and therefore regulates the number of X chromosomes to be eliminated. From a theoretical point of view, it is possible to develop a model with 1-factor to control the number of X chromosomes to be eliminated in the zygote [25]. This model contains genes (*e*), (*s*), and (*g*). Genes (*s*) and (*g*) have the same function as in the 2-factor model. Gene (*e*) has a maternal effect and regulates the number of X chromosomes eliminated in the zygote, as in the 2-factor model, but it has the ability to interact directly with the X chromosome in the zygote causing its elimination; the stoichiometry of the interaction is 1[e]:1[X] (Supplementary Material Text S4). This 1-factor model was analysed for the scenario where the imprinting gene (*g*) is fixed in the population and the gene (*s*) was under recessive positive selection to compare with the 2-factor model analysed above. It is only considered that gene (*e*) does not have a positive selective value; otherwise, it becomes homozygous in the population and this cannot reach the monogenic state.

**Figure 10:**
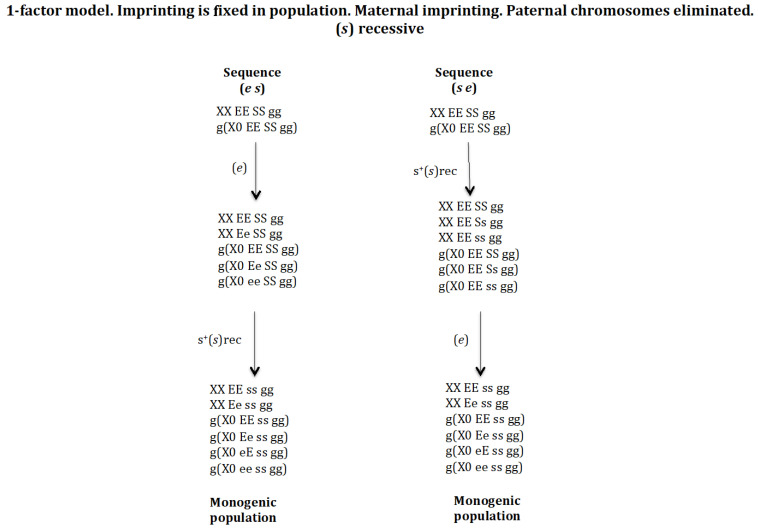
The 1-factor model. Imprinting is fixed in population. Maternal imprinting and paternal chromosomes are eliminated. (*s*) is recessive (rec). The stable states corresponding to the sequence (*e s*) are the following. Initial state *XX EE SS gg* = 1, *g*(*X0 EE SS gg*) = 1, SR = 1. Intermediate stable state corresponding to the emergence of (*e*) without positive selection is *XX EE SS gg* = 0.625, *XX Ee SS gg* = 0.375, *g*(*X0 EE SS gg*) = 0.375, *g*(*X0 Ee SS gg*) = 0.5, *g*(*X0 ee SS gg*) = 0.125, SR = 1.8. Final state of the evolutionary sequence (*e s*) corresponding to the emergence of (*s*) in the previous population without or with recessive positive selection for s^+^(*s*) *XX EE ss gg* = 0.5, *XX Ee ss gg* = 0.5, *g*(*X0 EE ss gg*) = 0.25, *g*(*X0 Ee ss gg*) = 0.25, *g*(*X0 eE ss gg*) = 0.25, *g*(*X0 ee ss gg*) = 0.25, SR = 1. The stable states corresponding to the sequence (*s*,*e*) are the following. Initial state *XX EE SS gg* = 1, *g*(*X0 EE SS gg*) = 1, SR = 1. Intermediate stable state corresponding to the emergence of (*s*) with recessive positive selection s^+^(*s*)rec *XX EE SS gg* = 0.157, *XX EE Ss gg* = 0.464, *XX EE ss gg* = 0.378, *g*(*X0 EE SS gg*) = 0.252, *g*(*X0 EE Ss gg*) = 0.524, *g*(*X0 EE ss gg*) = 0.224, SR = 0.62. Final state of the evolutionary sequence (*s e*) corresponding to the emergence of (*e*) in the previous population without positive selection *XX EE ss gg* = 0.5, *XX Ee ss gg* = 0.5, *g*(*X0 EE ss gg*) = 0.25, *g*(*X0 Ee ss gg*) = 0.25, *g*(*X0 eE ss gg*) = 0.25, *g*(*X0 ee ss gg*) = 0.25, SR = 1.

The initial digenic population was *XX EE SS gg* and *g*(*X0 EE SS gg*). Then, the genes (*e*) and (*s*) were introduced sequentially following sequences (*e s*) and (*s e*) under recessive positive selection for gene (*s*). The final state in both sequences is a monogenic population composed by the genotypes: *XX EE ss gg*, *XX Ee ss gg*, *g*(*X0 EE ss gg*), *g*(*X0 Ee ss gg*), *g*(*X0 eE ss gg*) and *g*(*X0 ee ss gg*) (Figure 10). *XX EE ss gg* is the gynogenic female because it does not carry the gene (*e*) and therefore the oocytes do not carry the elimination factor [e] and the zygotes do not eliminate any X chromosome. *XX Ee ss gg* is the androgenic female because it carries the gene (*e*) and the oocytes carry the factor 1[e] that interacts in the zygote with the paternally inherited X chromosome causing its elimination, whilst the maternally inherited X chromosome is immune to the factor [e] because it is imprinted.

## 4. Discussion

In this work, it has been demonstrated how the evolution of a monogenic population from a digenic one is possible under conditions of maternal imprinting and elimination of the paternal X chromosome in the zygote, as well as of the entire paternal chromosomal complement in spermatogenesis. The number and types of evolutionary transitions to the monogenic state depend on the dominant or recessive characteristics of the newly emerging genes (*e*), (*s*), and (*r*):

1. Under conditions of dominance for both (*s*) and (*r*), it is not possible to generate a monogenic population.
2. Under conditions of dominance for (*s*) and recessiveness for (*r*), it is possible to generate a monogenic population through the evolutionary transition sequence (*r s e*).
3. Under conditions of recessiveness for (*s*) and dominance for (*r*), it is not possible to evolve a monogenic population.
4. Under recessive conditions for both (*s*) and (*r*), a monogenic population can be generated by the evolutionary transition sequences (*r s e*), (*s r e*), and (*e s r*).
5. The evolution of a monogenic population is not possible in the absence of chromosomal imprinting.
6. The process of chromosomal imprinting must be present at the beginning of the evolutionary transition; that is, in the digenic population *XX EE SS RR gg* and *g*(*X0 EE SS RR gg*) for it to evolve into a monogenic population after the sequential appearance of the genes (*e*), (*s*), and (*r*).

The genetic basis described here for the evolution of a digenic population to a monogenic one considers that the genes (*e*), (*s*), (*r*), and (*g*) involved are located, for simplicity, on the autosomes. The monogenic population was determined to be composed of the two classes of females that characterise the monogenic trait of the population: *XX Ee ss rr gg* (androgenic females) and *XX ee ss rr gg* (gynogenic females), in addition to the males *g*(*X0 EE ss rr gg*), *g*(*X0 Ee ss rr gg*), *g*(*X0 eE ss rr gg*), and *g*(*X0 ee ss rr gg*). As a rule, the two females have an equal frequency of 50% and the males a frequency of 0.25 and the sex ratio (males/females) is 1. The androgenic female produces oocytes carrying one dose of the maternal factor 1[e], which inhibits, in the zygote, one dose of the elimination factor 1[r], so that the remaining factor 1[r] interacts with the paternally derived X chromosome causing its elimination. However, the remaining maternal X chromosome is not eliminated, since it is imprinted (protected) from the inhibitory effect of the elimination factor [r]. Therefore, androgenic females produce only males carrying the maternal X chromosome. The gynogenic female produces oocytes carrying two doses of the maternal factor 2[e], so that in the zygote, both doses of the elimination factor 2[r] are inactivated and no X chromosome elimination occurs, these zygotes retaining both the maternal and paternal X chromosomes. The genetic basis that characterises a monogenic population is explained below. To this purpose, chromosomes and gametes will be identified by the letters “m” and “p” to indicate their origin, whether maternal or paternal, respectively. This will only apply to the X chromosome, since it is the only that is eliminated. It should be remembered that the stoichiometry of the interaction between the elimination factor [r] and its maternal inhibitor [e] is 1:1 and that homozygosis of the gene (*s*) determines that only chromosomes of maternal origin will form the sperm.

### 4.1. The Case of the 2-Factor Model. Generation of Androgenic Females (Figure 11)

**Figure 11:**
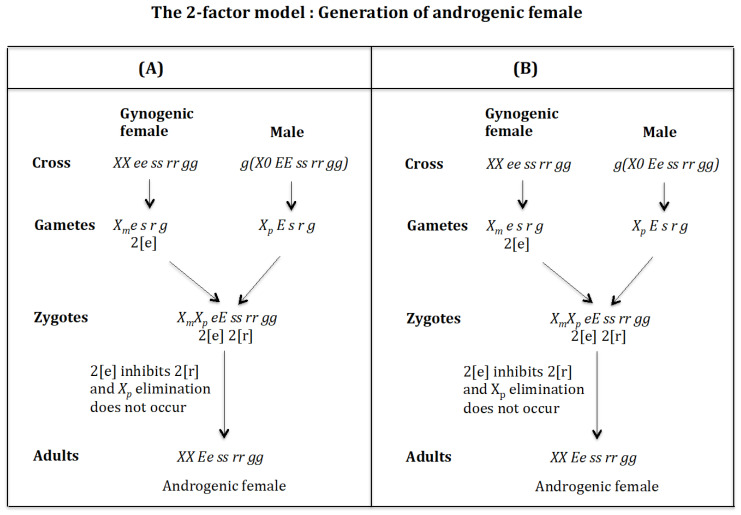
The 2-factor model: Generation of androgenic females. (**A**) Cross of female *XX ee ss rr gg* with male g(X0 EE ss rr gg). (**B**) Cross of female XX ee ss rr gg with male g(X0 Ee ss rr gg).

**Cross of female *XX ee ss rr gg* with male** g(X0 EE ss rr gg). (Figure 11A). The female produces oocytes $X_{m}e\;s\;r\;g$ containing two maternal factors 2[e]. The male produces sperm $X_{p}E\;s\;r\;g$ and none *0 E s r g* owing to homozygosis for gene (*s*). The zygote is $X_{m}X_{p}\;eE\;ss\;rr\;gg$. Factor 2[e] inhibits factor 2[r] and the $X_{p}$ chromosome is not eliminated. The result is the formation of the androgenic female *XX Ee ss rr gg*.

**Cross of female** XX ee ss rr gg **with male** g(X0 Ee ss rr gg)**.** (Figure 11B). The female produces oocytes $X_{m}e\;s\;r\;g$ containing two maternal factors 2[e]. The male produces sperm $X_{p}E\;s\;r\;g$ and none *0 e s r g* owing to homozygosis for gene (*s*). The zygote is $X_{m}X_{p}\;eE\;ss\;rr\;gg$. Factor 2[e] inhibits factor 2[r] and the $X_{p}$ chromosome is not eliminated. The result is the formation of the androgenic female *XX Ee ss rr gg*.

### 4.2. The Case of the 2-Factor Model. Generation of Gynogenic Females (Figure 12)

**Figure 12:**
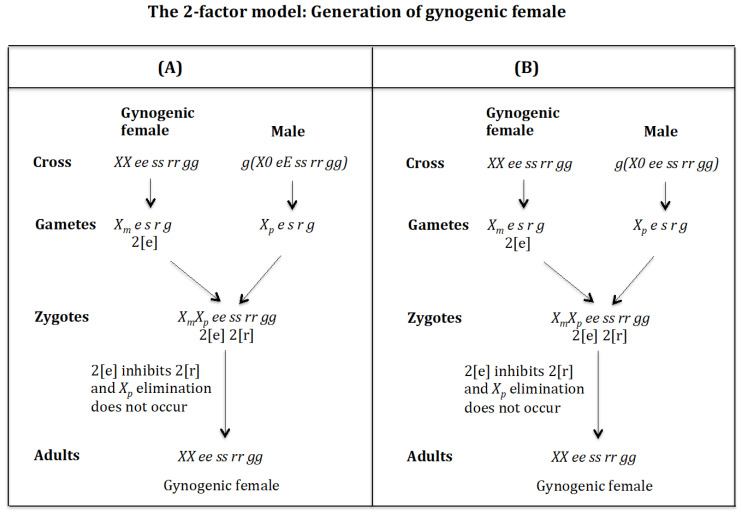
The 2-factor model: Generation of androgenic females. (**A**) Cross of female XX ee ss rr gg with male g(X0 eE ss rr gg). (**B**) Cross of female XX ee ss rr gg with male g(X0 ee ss rr gg).

**Cross of female** XX ee ss rr gg **with male** g(X0 eE ss rr gg). (Figure 12A). The female produces oocytes $X_{m}e\;s\;r\;g$ containing two maternal factors 2[e]. The male produces sperm $X_{p}e\;s\;r\;g$ and none *0 E s r g* owing to homozygosis for gene (*s*). The zygote is $X_{m}X_{p}\;ee\;ss\;rr\;gg$. Factor 2[e] inhibits factor 2[r] and the $X_{p}$ chromosome is not eliminated. The result is the formation of the gynogenic female *XX ee ss rr gg*.

**Cross of female** XX ee ss rr gg **with male** g(X0 ee ss rr gg). (Figure 12B). The female produces oocytes $X_{m}e\;s\;r\;g$ containing two maternal factors 2[e]. The male produces sperm $X_{p}e\;s\;r\;g$ and none *0 e s r g* owing to homozygosis for gene (*s*). The zygote is $X_{m}X_{p}\;ee\;ss\;rr\;gg$. Factor 2[e] inhibits factor 2[r] and the $X_{p}$ chromosome is not eliminated. The result is the formation of the gynogenic female *XX ee ss rr gg*.

### 4.3. The Case of the 2-Factor Model. Generation of Males (Figure 13 and Figure 14)

**Figure 13:**
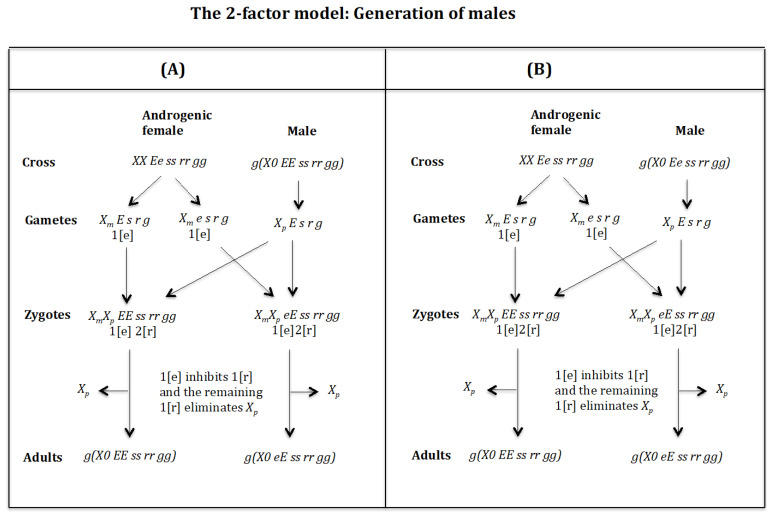
The 2-factor model. (**A**) Cross of female XX Ee ss rr gg with male g(X0 EE ss rr gg). (**B**) Cross of female XX Ee ss rr gg with male g(X0 Ee ss rr gg).

**Cross of female** XX Ee ss rr gg **with male** g(X0 EE ss rr gg). (Figure 13A). The female produces oocytes $X_{m}E\;s\;r\;g$ and $X_{m}e\;s\;r\;g$ both containing maternal factor 1[e]. The male produces sperm $X_{p}E\;s\;r\;g$ and none *0 E s r g* owing to homozygosis for gene (*s*). The zygotes are $X_{m}X_{p}\;EE\;ss\;rr\;gg$ and $X_{m}X_{p}\;eE\;ss\;rr\;gg$, which develop, respectively, into adult males $g(X_{m}0\;EE\;ss\;rr\;gg)$ and ${g(X}_{m}0\;eE\;ss\;rr\;gg)$ because factor 1[e] inhibits factor 1[r] and the remaining factor 1[r] causes the elimination of the $X_{p}$, whilst the $X_{m}$ being imprinted is unaffected by the elimination factor [r]. The result is the formation of males g(X0 EE ss rr gg) and g(X0 eE ss rr gg).

**Figure 14:**
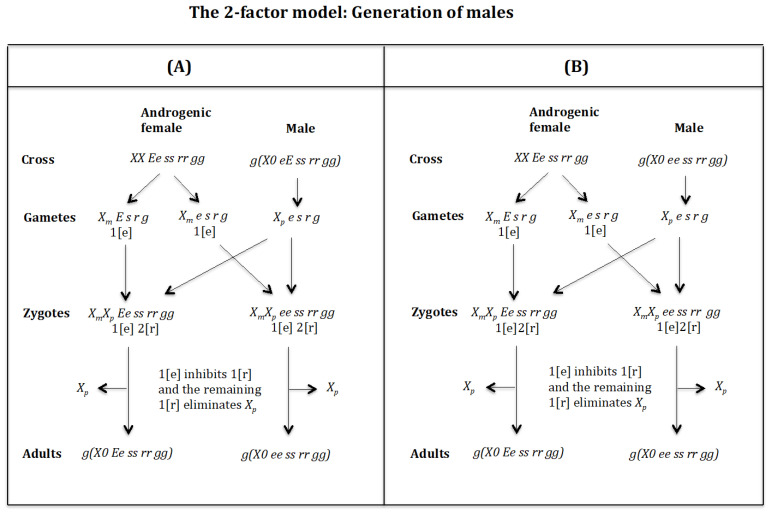
The 2-factor model. Generation of males. (**A**) Cross of female XX Ee ss rr gg with male g(X0 eE ss rr gg). (**B**) Cross of female XX Ee ss rr gg with male g(X0 ee ss rr gg).

**Cross of female** XX Ee ss rr gg **with male** g(X0 Ee ss rr gg). (Figure 13B). The female produces oocytes $X_{m}E\;s\;r\;g$ and $X_{m}e\;s\;r\;g$ both containing maternal factor 1[e]. The male produces sperm $X_{p}E\;s\;r\;g$ and none *0 e s r g* owing to homozygosis for gene (*s*). The zygotes are $X_{m}X_{p}\;EE\;ss\;rr\;gg$ and $X_{m}X_{p}\;eE\;ss\;rr\;gg$, which develop, respectively, into adult males $g(X_{m}0\;EE\;ss\;rr\;gg)$ and ${g(X}_{m}0\;eE\;ss\;rr\;gg)$ because factor 1[e] inhibits factor 1[r] and the remaining factor 1[r] causes the elimination of $X_{p}$, whilst $X_{m}$ being imprinted is not affected by the elimination factor [r]. The result is the formation of males g(X0 EE ss rr gg) and g(X0 eE ss rr gg).

**Cross of female** XX Ee ss rr gg **with male** g(X0 eE ss rr gg)**.** (Figure 14A). The female produces oocytes $X_{m}E\;s\;r\;g$ and $X_{m}e\;s\;r\;g$ both containing maternal factor 1[e]. The male produces sperm $X_{p}e\;s\;r\;g$ and none *0 E s r g* owing to homozygosis for gene (*s*). The zygotes are $X_{m}X_{p}\;Ee\;ss\;rr\;gg$ and $X_{m}X_{p}\;ee\;ss\;rr\;gg$, which develop, respectively, into adult males g(X0 Ee ss rr gg) and g(X0 ee ss rr gg) because factor 1[e] inhibits factor 1[r] and the remaining factor 1[r] causes the elimination of $X_{p}$, whilst $X_{m}$ being imprinted is not affected by the elimination factor [r]. The result is the formation of males g(X0 Ee ss rr gg) and g(X0 ee ss rr gg).

**Cross of female** XX Ee ss rr gg **with male** g(X0 ee ss rr gg)**.** (Figure 14B). The female produces oocytes $X_{m}E\;s\;r\;g$ and $X_{m}e\;s\;r\;g$ both containing maternal factor 1[e]. The male produces sperm $X_{p}e\;s\;r\;g$ and none *0 e s r g* owing to homozygosis for gene (*s*). The zygotes are $X_{m}X_{p}\;Ee\;ss\;rr\;gg$ and $X_{m}X_{p}\;ee\;ss\;rr\;gg$, which develop, respectively, into adult males $g(X_{m}0\;Ee\;ss\;rr\;gg)$ and ${g(X}_{m}0\;ee\;ss\;rr\;gg)$ because factor 1[e] inhibits factor 1[r] and the remaining factor 1[r] causes the elimination of $X_{p}$, whilst $X_{m}$ being imprinted is not affected by the elimination factor [r]. The result is the formation of males g(X0 Ee ss rr gg) and g(X0 ee ss rr gg).

### 4.4. The Case of the 1-Factor Model. Generation of Gynogenic Females (Figure 15)

**Figure 15:**
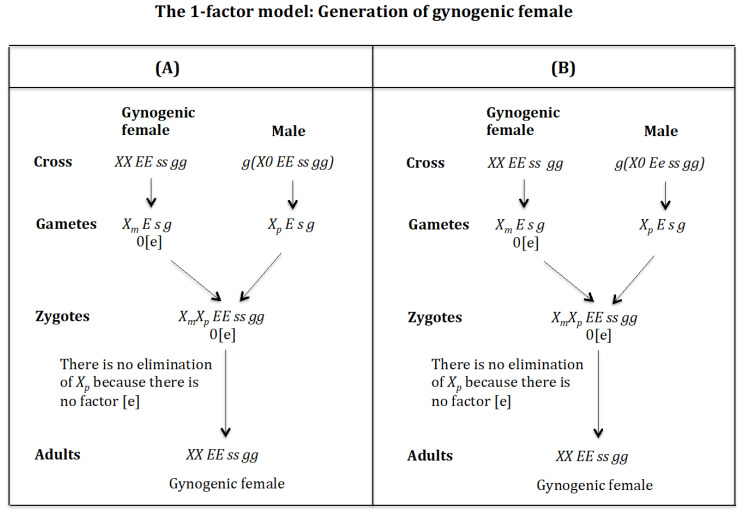
The 1-factor model. Generation of gynogenic females. (**A**) Cross of female XX EE ss gg with male g(X0 EE ss gg). (**B**) Cross of female XX EE ss gg with male g(X0 Ee ss gg).

**Generation of gynogenic females.** (Figure 15A). **Cross of female** XX EE ss gg **with male** g(X0 EE ss gg). The female produces oocytes $X_{m}E\;s\;g$ without the maternal factor [e]. The male produces sperm $X_{p}E\;s\;g$ and none *0 E s g* owing to homozygosis for gene (*s*). The zygote is $X_{m}X_{p}\;EE\;ss\;gg$. The lack of factor [e] determines that the $X_{p}$ chromosome is not eliminated. The result is the formation of the gynogenic female *XX EE ss gg*.

**Generation of gynogenic females** (Figure 15B). **Cross of female** XX EE ss gg **with male** g(X0 Ee ss gg). The female produces oocytes $X_{m}E\;s\;g$ without the maternal factor [e]. The male produces sperm $X_{p}E\;s\;g$ and none *0 e s g* owing to homozygosis for gene (*s*). The zygote is $X_{m}X_{p}\;EE\;ss\;gg$. The lack of factor [e] determines that the $X_{p}$ chromosome is not eliminated. The result is the formation of the gynogenic female *XX EE ss rr gg*.

### 4.5. The Case of the 1-Factor Model. Generation of Androgenic Females (Figure 16)

**Figure 16:**
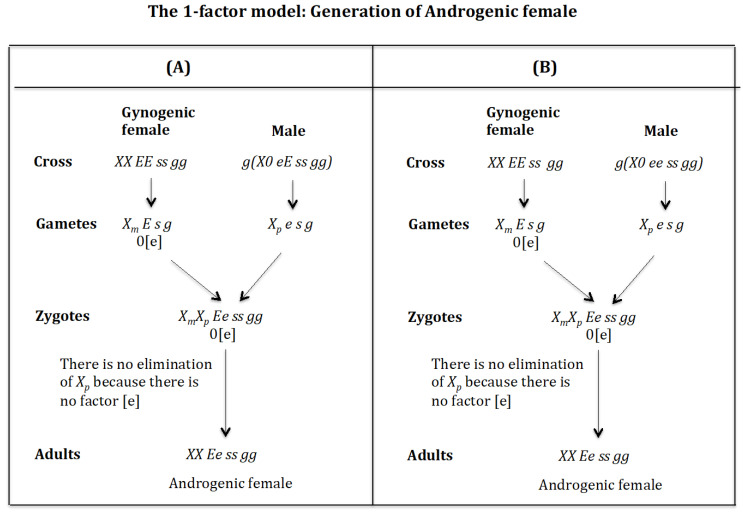
The 1-factor model. Generation of androgenic females. (**A**) Cross of female XX EE ss gg with male g(X0 eE ss gg). (**B**) Cross of female XX EE ss gg with male g(X0 ee ss gg).

**Generation of androgenic females** (Figure 16A). **Cross of female** XX EE ss gg **with male** g(X0 eE ss gg). The female produces oocytes $X_{m}E\;s\;g$ without the maternal factor [e]. The male produces sperm $X_{p}e\;s\;g$ and none *0 E s g* owing to homozygosis for gene (*s*). The zygote is $X_{m}X_{p}\;Ee\;ss\;gg$. The lack of factor [e] determines that the $X_{p}$ chromosome is not eliminated. The result is the formation of the androgenic female *XX Ee ss rr gg*.

**Generation of androgenic females** (Figure 16B). **Cross of female** XX EE ss gg **with male** g(X0 ee ss gg). The female produces oocytes $X_{m}E\;s\;g$ without the maternal factor [e]. The male produces sperm $X_{p}e\;s\;g$ and none *0 e s g* owing to homozygosis for gene (*s*). The zygote is $X_{m}X_{p}\;Ee\;ss\;gg$. The lack of factor [e] determines that the $X_{p}$ chromosome is not eliminated. The result is the formation of the androgenic female *XX Ee ss rr gg*.

### 4.6. The Case of the 1-Factor Model. Generation of Males (Figure 17 and Figure 18)

**Figure 17:**
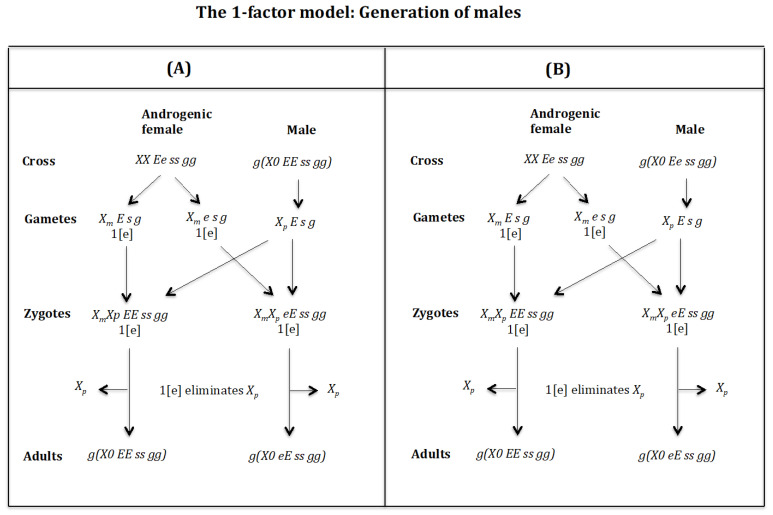
The 1-factor model. Generation of males. (**A**) Cross of female XX Ee ss gg with male g(X0 EE ss gg). (**B**) Cross of female XX Ee ss gg with male g(X0 Ee ss gg).

**Generation of males** (Figure 17A). **Cross of female** XX Ee ss gg **with male** g(X0 EE ss gg). The female produces oocytes $X_{m}E\;s\;g$ and $X_{m}e\;s\;g$ with one dose of maternal factor 1[e]. The male produces sperm $X_{p}E\;s\;g$ and none *0 E s g* owing to homozygosis for gene (*s*). The zygotes are $X_{m}X_{p}\;EE\;ss\;gg$ and $X_{m}X_{p}\;eE\;ss\;gg.$ Factor 1[e] interacts with the $X_{p}$ chromosome causing its elimination giving rise to, respectively, the adults *g*(*X0 EE ss gg*) and *g*(*X0 eE ss gg*). Factor [e] does not interact with the $X_{m}$ chromosome because it is imprinted.

**Figure 18:**
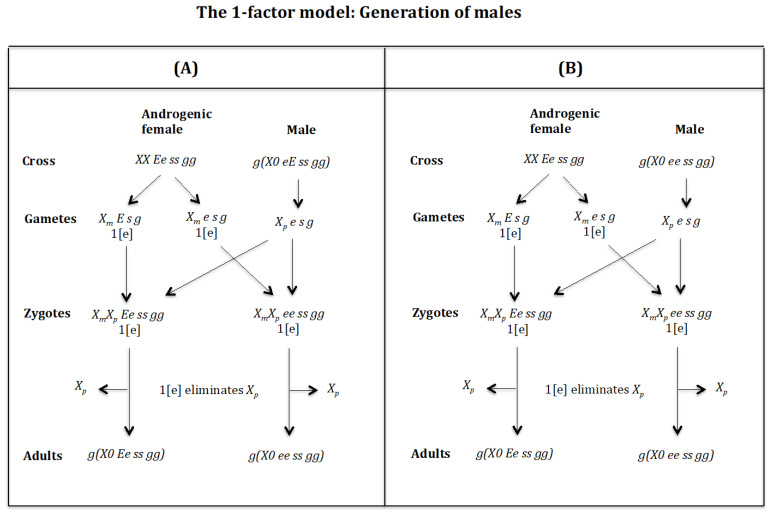
The 1-factor model. Generation of males. (**A**) Cross of female XX Ee ss gg with male g(X0 eE ss gg). (**B**) Cross of female XX Ee ss gg with male g(X0 ee ss gg).

**Generation of males** (Figure 17B). **Cross of female** XX Ee ss gg **with male** g(X0 Ee ss gg). The female produces oocytes $X_{m}E\;s\;g$ and $X_{m}e\;s\;g$ with one dose of maternal factor 1[e]. The male produces sperm $X_{p}E\;s\;g$ and none *0 E s g* owing to homozygosis for gene (*s*). The zygotes are $X_{m}X_{p}\;EE\;ss\;gg$ and $X_{m}X_{p}\;eE\;ss\;gg.$ Factor 1[e] interacts with the $X_{p}$ chromosome causing its elimination giving rise to, respectively, the adults *g*(*X0 EE ss gg*) and *g*(*X0 eE ss gg*). Factor [e] does not interact with the $X_{m}$ chromosome because it is imprinted.

**Generation of males** (Figure 18A). **Cross of female** XX Ee ss gg **with male**g(X0 eE ss gg). The female produces oocytes $X_{m}E\;s\;g$ and $X_{m}e\;s\;g$ with one dose of maternal factor 1[e]. The male produces sperm $X_{p}e\;s\;g$ and none *0 E s g* owing to homozygosis for gene (*s*). The zygotes are $X_{m}X_{p}\;Ee\;ss\;gg$ and $X_{m}X_{p}\;ee\;ss\;gg.$ Factor 1[e] interacts with the $X_{p}$ chromosome causing its elimination giving rise to, respectively, the adults *g*(*X0 Ee ss gg*) and *g*(*X0 ee ss gg*). Factor [e] does not interact with the $X_{m}$ chromosome because it is imprinted.

**Generation of males** (Figure 18B). **Cross of female** XX Ee ss gg **with male**g(X0 ee ss gg). The female produces oocytes $X_{m}E\;s\;g$ and $X_{m}e\;s\;g$ with one dose of maternal factor 1[e]. The male produces sperm $X_{p}e\;s\;g$ and none *0 E s g* owing to homozygosis for gene (*s*). The zygotes are $X_{m}X_{p}\;Ee\;ss\;gg$ and $X_{m}X_{p}\;ee\;ss\;gg.$ Factor 1[e] interacts with the $X_{p}$ chromosome causing its elimination giving rise to, respectively, the adults *g*(*X0 Ee ss gg*) and *g*(*X0 ee ss gg*). Factor [e] does not interact with the $X_{m}$ chromosome because it is imprinted.

The genetic basis described here for monogenic species can be applied to the monogenic species such as Cecydomyiidae insects (gall midges), where all zygotes start out with the X_1_X_1_X_2_X_2_ constitution and develop as female, whereas if two X chromosomes (X_1_X_2_) are deleted, the embryo becomes X_1_X_2_0 and develops as male [9,10,11]. A similar situation is found in the Collembola insects (springtails), where the zygotes start with the XXXX constitution. If the embryo does not delete any X chromosome, it remains XXXX and develops as female, whereas if two X chromosomes are deleted, the embryo becomes XX0 and develops as male [12,13,14].

It was found that in some evolutionary lineages, from the digenic state to the monogenic state, the latter state is not reached, but the population becomes extinct due to a lack of males. To explain this, let us analyse a simple case: the sequence (*s g r*) in “Putative acquisition of chromosomal imprinting” (Figure 8). It was considered that the gene (*s*) first arose in the population *XX EE SS RR GG* and *X0 EE SS RR GG*, giving rise to population *XX EE ss RR GG* and *X0 EE ss RR GG*. The appearance of the gene (*g*) in that population causes its extinction owing to a lack of males. This is because the parental genotypes *XX EE ss RR GG* and *X0 EE ss RR GG*, as well as the intermediate genotypes *XX EE ss RR Gg*, *X0 EE ss RR GG*, *X0 EE ss RR Gg*, *X0 EE ss RR gG* and *g*(*X0 EE ss RR gg*) generated after the appearance of gene (*g*) in the population, are eliminated over the generations, until a state composed exclusively of *XX EE ss RR gg* females and *g*(*X0 EE ss RR gG*) males is reached. This state marks the end of the population, since crosses between these females and these males only produce females and not males, since the males *g*(*X0 EE ss RR gG*) produce only *X E s R g* sperm, which carry the maternal chromosome set, being homozygous for gene (*s*). The same explanation applies to the other cases of population extinction, with the sole exception that in the case of the sequence (*e s*), in Figure 3, the population is composed of *XX EE ss RR gg*, *XX Ee ss RR gg*, *XX ee ss RR gg*, *g*(*X0 EE sS RR gg*) and *g*(*X0 eE sS RR gg*); and in the case of sequence (*r s*), in Figure 3, the population is *XX EE ss RR gg* and *g*(*X0 eE sS RR gg*).

It was found that the evolution of a monogenic population from a digenic one is not possible in the absence of chromosomal imprinting, which operates in the mother, marking the X chromosome during oogenesis, thus preventing the inhibitory effect of the elimination factor [r] in the zygote, in the case of the 2-factor model, or the inhibitory effect of the elimination factor [e], in the case of the 1-factor model. It was determined here that the gene (*g*) responsible for the imprinting process, regardless of its molecular nature, must already be fixed (homozygous) in the digenic population from which the monogenic population evolves; otherwise, the population cannot reach the monogenic state. This does not necessarily mean that the original digenic population had an imprinting process for sex determination. It could well be that the original population had such a process involved in a different biological process, if it existed, and that it was co-opted when the genes (*e*), (*s*) and (*r*) begin to emerge in the population.

Finally, the monogenic state of the population had still some plasticity to continue evolving and maintain its monogenic characteristic. The monogenic population *XX Ee ss rr gg* (androgenic female), *XX ee ss rr gg* (gynogenic females), *g*(*X0 EE ss rr gg*), *g*(*X0 Ee ss rr gg*), *g*(*X0 eE ss rr gg*) and *g*(*X0 ee ss rr gg*) has a capacity to evolve further towards the monogenic population XX Ee* ss rr gg (gynogenic), *XX EE ss rr gg* (androgenic) and *g*(*X0 EE ss rr gg*) if the gene (*e*) mutates towards a new allele (e*) whose encoded product has a higher inhibitory efficiency in the elimination factor [r] becoming the stoichiometry of the inhibition 1[e*]:2[r]. This is understandable, since it is the number of X chromosomes eliminated that ultimately determines the existence of two classes of females: those that produce only females and those that produce only males, characteristic of a monogenic population.

The sex ratio of *Sciara ocellaris* varies widely from progenies with few males to progenies containing a larger proportion of males, with single-sex progenies being rare. The sex-ratio distributions were dependent on the temperature at which the stocks of flies were raised, with the sex-ratio distributions being symmetrical (about 50% males) at 18 °C and 20 °C while at the higher temperatures of 24 °C and 28 °C, the distributions were skewed toward a high proportion of females with the proportion of males decreasing to about 30–37% per progeny. Temperature-shift experiments showed that high temperatures were effective only during the last stages of female pupal development plus a period after adult emergence, stages corresponding to oocyte maturation [26]. The 2-factor and 1-factor models proposed here can explain these results. The thermo-sensitive period corresponds to the moment in which gene (*e*) is expressed and its encoded product is stored in the oocyte. The expression of gene (*e*) in *S. ocellaris* would vary between females, so that the functional threshold amount of product [e] accumulated in the oocyte would vary between them. This variation would be smaller at high than at low temperatures, with the amount of product accumulated in the oocyte being more similar between females. This would determine that, at high temperatures, more females than males would be produced.

### 4.7. The 2-Factor Model Versus the 1-Factor Model

Both models can theoretically explain the evolution of monogenic populations from digenic populations. An experimental intervention could help distinguish between the two models: UV-irradiation of embryos at the syncytial blastoderm stage [27,28,29,30]. UV-irradiation is considered to cause destruction of the maternal factor [e].

Following the 2-factor model, local destruction of the maternal factor [e] in UV-irradiated embryos of female-producer gynogenic females *X_m_X_p_ ee ss rr gg* would determine that in some cells factor [r] would not be inhibited and, therefore, would interact with the paternally inherited *Xp* chromosome, causing its elimination. If this happens, the adult female carries patches of *X_m_0* male constitution; that is, a gynandromorph is generated. If UV-treatment destroyed factor [r], *X_p_* elimination would not occur in some cells and these would remain as female cells, indistinguishable from the rest of cells. In the case of the 1-factor model, UV-irradiation treatment is not expected to interfere with X chromosome elimination, since *X_m_X_p_ EE ss gg* gynogenic females do not carry the gene (*e*), so gynandromorphs would not be produced.

Following the 2-factor model, local destruction of maternal factor [e] in UV-irradiated embryos of male-producer androgenic females *X_m_X_p_ Ee rr ss gg* would determine that some factor [r] would not be inhibited in some cells and the *X_p_* chromosome would be eliminated in those cells whilst the *X_m_* chromosome would not be eliminated because it is imprinted. The result is the production of male *X0* patches, which cannot be distinguished from the rest of the male cells. In the case of 1-factor model, in UV-irradiated embryos from male-producer androgenic females *XX Ee ss gg*, local destruction of factor [e] prevents the elimination of the *X_p_* chromosome in some cells, which remain *X_m_X_p_*, resulting in a male with female spots (gynandromorph).

In summary, the 2-factor model predicts the possible generation of gynandromorphs after UV-irradiation of embryos from gynogenic females, which is not expected in the 1-factor model. The 1-factor model predicts the possible generation of gynandromorphs after UV-irradiation of embryos from androgenic females, which is not expected in the 2-factor model. From an evolutionary perspective, both models could exist in nature in different insects. As for their origin, they either resulted from two independent events or there was an evolutionary relationship between them.

### 4.8. The Particular Case of Sciara (Figure 19 and Figure 20)

The two classes of *Sciara* females, gynogenic and androgenic, differ in the existence of a special X chromosome (*X′*) in gynogenic females, which are *X′X*, whereas androgenic females are *XX*. *X0* males do not carry the *X′* chromosome [8,31,32,33]. There is an inversion on the *X′* chromosome that prevents its recombination with the homologous X chromosome, thus retaining the *X′* factor for female production [34]. The molecular characterisation of chromosome *X′* has been analysed. It was found that the inversion spans most of the X′ chromosome (about 55 Mb) and encodes approximately 3500 genes. Analysis of the divergence between the inversion and the homologous region of the X revealed that it originated very recently (<0.5 Ma). It was found that the X′ is more complex than previously thought and is likely to have undergone multiple rearrangements that have produced regions of varying ages, resembling a supergene composed of evolutionary strata. We found functional degradation of approximately 7.3% of genes within the region of recombination suppression, but no evidence of accumulation of repetitive elements [35]. According to the nomenclature used here, the *X′* chromosome corresponds to the one that carries the gene (*e*), which encodes the maternal factor that inhibits the elimination factor [r] in the zygote. Therefore, in *Sciara*, the females *X′X* would be ${Inv(X}_{e})X\;$(gynogenic) and *XX* would be androgenic. Furthermore, *Sciara* also has the particularity that the zygote contains 3X chromosomes: the oocyte contributes an X chromosome and the sperm contributes 2X chromosomes corresponding to the two chromatids of the maternal X chromosome that remain attached at the end of spermatogenesis [8]. The genetic model developed here can also be applied to *Sciara* with the following additional considerations: the gene (*e*) is located on the *X* chromosome and is only expressed in females during oogenesis and its product, the factor [e], is stored in the oocyte; in addition, it is necessary to include a new gene (*c*), responsible for the non-disjunction of the two chromatids of the maternal *X* chromosome during spermatogenesis.

**Figure 19:**
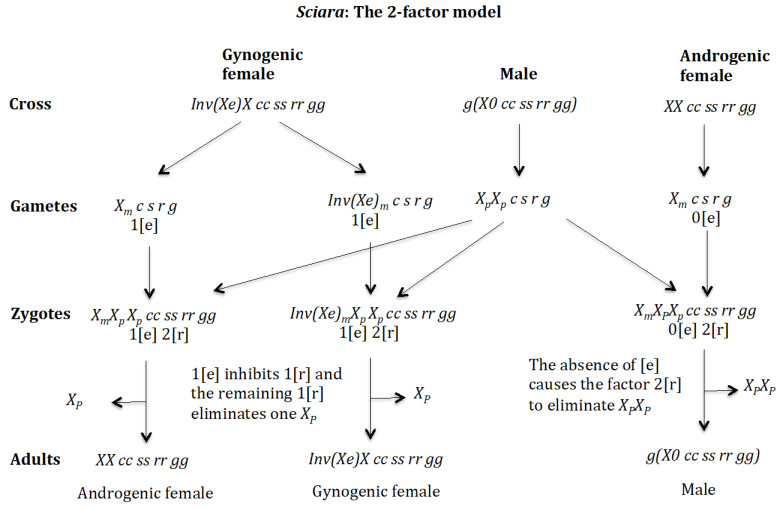
*Sciara*. The 2-factor model. Cross of gynogenic female $Inv\left(X_{e}\right)X\;cc\;ss\;rr\;gg$ and androgenic female XX cc ss rr gg with male g(X0 cc ss rr gg).

**Figure 20:**
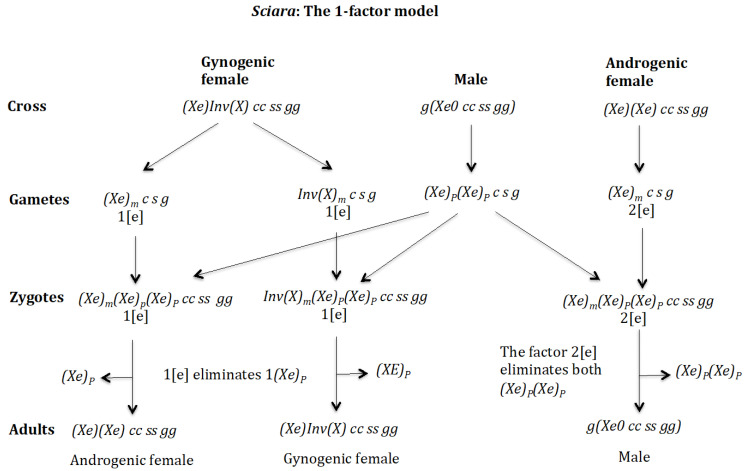
*Sciara*. The 1-factor model. Cross of gynogenic female Xe*Inv*(*X*) *cc ss rr gg* and androgenic female (Xe)(Xe) cc ss rr gg with male *g*(Xe*0 cc ss rr gg*).

Following the 2-factor model, the genotypic formula proposed here for sex determination in *Sciara* is: female $Inv\left(X_{e}\right)X\;cc\;ss\;rr\;gg\;$(gynogenic), female XX cc ss rr gg (androgenic) and male g(X0 cc ss rr gg). Gene (*c*) is located in an autosome for simplicity. Chromosome $Inv\left(X_{e}\right)$ corresponds to the *X′* chromosome described above, which carries an inversion.

**The 2-factor model** (Figure 19). **Cross of gynogenic female** $Inv\left(X_{e}\right)X\;cc\;ss\;rr\;gg$ **with male** g(X0 cc ss rr gg). The female produces oocytes *Inv*(*X_e_*)*_m_ c s r g* and *X_m_ c s r g* both containing maternal factor 1[e]. The male produces only sperm *X_p_X_p_ c s r g* because it is homozygous for gene (*c*) and none sperm *0 c s r g* because it is homozygous for (*s*). The zygotes are *X_m_X_p_ X_p_ cc ss rr gg* and *Inv*(*X_e_*)*_m_X_p_X_p_ cc ss rr gg*. In both cases, factor 1[e] inhibits factor 1[r] and the remaining factor 1[r] eliminates only one *X_p_* and the zygotes, respectively, develop into adult females $XX\;cc\;ss\;rr\;gg\left(androgenic\right)$ and $Inv\left(X_{e}\right)X\;cc\;ss\;rr\;gg(gynogenic)$. The chromosomes *X_m_* and *Inv*(*X_e_*)*_m_* are not eliminated because they are imprinted. **Cross of androgenic female** XX cc ss rr gg **with male** g(X0 cc ss rr gg). The female produces oocytes $X_{m}\;c\;s\;r\;g$ that do not contain the maternal factor [e]. The male produces only sperm *X_p_X_p_ c s r g* because it is homozygous for gene (*c*) and none sperm *0 c s r g* because it is homozygous for (*s*). In the zygotes $X_{m}X_{p}X_{p}\;cc\;ss\;rr\;gg$, lacking the maternal factor [e], the elimination factor 2[r] delete both $X_{p}X_{p}$ chromosomes and become adult males g(X0 cc ss rr gg).

In the case of the 1-factor model, the following genotypic formula is proposed for sex determination in *Sciara*: female $XeInv\left(X\right)\;cc\;ss\;gg$ (gynogenic), female (Xe)(Xe) cc ss gg (androgenic) and male g(Xe0 cc ss gg). The gene (*c*) is located in an autosome for simplicity. The $Inv\left(X\right)$ chromosome corresponds to the X′ chromosome described above, which carries an inversion. Note that in this case the inverted chromosome would not carry the gene (*e*) that controls the number of deleted X chromosomes in the embryo. However, it is shown below that the 1-factor model can also explain the genetic basis of sex determination in *Sciara*.

**The 1-factor model** (Figure 20)**. Cross of gynogenic female** $XeInv\left(X\right)cc\;ss\;gg$ **with male** g(Xe0 cc ss gg). The female produces oocytes ${\left(Xe\right)}_{m\;}c\;s\;g$ and $Inv{\left(X\right)}_{m\;}c\;s\;g$ both containing maternal factor 1[e]. The male produces only sperm $\left(Xe\right)$*_p_*$\left(Xe\right)$*_p_ c s g* because it is homozygous for gene (*c*) and none sperm *0 c s g* because it is homozygous for (*s*). The zygotes are $I{nv\left(X\right)}_{m}{\left(Xe\right)}_{p}{\left(Xe\right)}_{p}\;cc\;ss\;gg$ and ${\left(Xe\right)}_{m}{\left(Xe\right)}_{p}{\left(Xe\right)}_{p}\;cc\;ss\;gg$. In both cases, factor 1[e] eliminates only one $\left(Xe\right)$*_p_* and the zygotes, respectively, develop into adult females $\left(Xe\right)\left(Xe\right)\;cc\;ss\;gg\;\left(androgenic\right)\;and\;Inv\left(X\right)(Xe)\;cc\;ss\;gg\left(gynogenic\right)$. The chromosome ${\left(Xe\right)}_{m}$ is not eliminated because it is imprinted. **Cross of androgenic female** (Xe)(Xe)cc ss gg **with male** g(Xe0 cc ss gg). The female produces oocytes $({Xe)}_{m}c\;s\;g$ that contains the maternal factor 2[e]. The male produces sperm ${\left(Xe\right)}_{p}{\left(Xe\right)}_{p}c\;s\;g$ because it is homozygous for gene (*c*) and none sperm *0 c s g* because it is homozygous for (*s*). The zygotes are ${\left(Xe\right)}_{m}{\left(Xe\right)}_{p}{\left(Xe\right)}_{p}cc\;ss\;gg$. The factor 2[e] eliminates both ${\left(Xe\right)}_{p}{\left(Xe\right)}_{p}$ giving rise to the adult males g(Xe0 cc ss gg).

Thus, the X′ chromosome continues controlling the number of X chromosomes eliminated in the zygote of *Sciara*, as proposed by Metz [8]. This is because the female carrying the X′ chromosome produces oocytes with factor 1[e], whereas androgenic females will produce oocytes with factor 2[e].

What results are expected from UV-irradiation treatments of embryos according to the two models proposed for sex determination in *Sciara*?

According to the 2-factor model, UV-irradiation of embryos *X_m_X_p_ X_p_ cc ss rr gg* and Inv(Xe)XpXp cc ss rr gg, from the cross between $Inv\left(X_{e}\right)X\;cc\;ss\;rr\;gg$ with male g(X0 cc ss rr gg), would cause local destruction of the maternal factor 1[e] and, consequently, the elimination factor 2[r] will interact with the *X_p_ X_p_* chromosomes causing their elimination. The result is the production of gynandromorphs carrying male cells g(*X0 cc ss rr gg*) or *g*(*Inv*(*Xe*)*0 cc ss rr gg*), respectively. UV-irradiation of embryos from androgenic females $X_{m}X_{p}X_{p}\;cc\;ss\;rr\;gg$ would not cause any effect because the embryos do not contain the maternal factor [e] and consequently those embryos would develop into normal $g\left(X0\;cc\;ss\;rr\;gg\right)$ males.

According to the 1-factor model, UV-irradiation of embryos ${\left(Xe\right)}_{m}{\left(Xe\right)}_{p}{\left(Xe\right)}_{p}\;cc\;ss\;gg$ and $I{nv\left(X\right)}_{m}{\left(Xe\right)}_{p}{\left(Xe\right)}_{p}\;cc\;ss\;gg$ from a cross between $(Xe)Inv\left(X\right)\;cc\;ss\;gg$ with male g(Xe0 cc ss gg) would cause local destruction of the maternal factor 1[e] and, consequently, no elimination of the $X_{p}X_{p}$ chromosomes will occur in both embryos, thus generating cells that become triploid for the X chromosome and probably lethal. UV-irradiation of embryos ${\left(Xe\right)}_{m}{\left(Xe\right)}_{p}{\left(Xe\right)}_{p}cc\;ss\;gg$ from the cross of androgenic females (Xe)(Xe) cc ss gg with male g(Xe0 cc ss gg), would destroy the factor 2[e] and, consequently, the elimination of the ${\left(Xe\right)}_{p}{\left(Xe\right)}_{p}$ chromosomes would not occur, the cells remaining triploid for the X chromosome and probably lethal.

In summary, the 2-factor model predicts that UV-irradiation of embryos from gynogenic females can produce females with male spots (gynandromorphs), a result not expected by the 1-factor model.

Regarding theoretical evolution of the sex determination mechanism in *Sciara*, it remains to be determined whether it could have its origin in one of the theoretical models discussed above or, alternatively, whether it represents an independent evolutionary lineage. In the first case, the following evolutionary events must have occurred: (1) the translocation of the gene (*e*) from an autosome to the X chromosome, and (2) the appearance of a new gene, gene (*c*), responsible for the non-disjunction of the two chromatids of the maternally inherited X chromosome during spermatogenesis.

## 5. Conclusions

A theoretical model is presented here that explains the evolutionary transition from a digenic to a monogenic population. A controlling gene was associated with each of the four processes that characterise monogenic populations: (1) gene (*s*) controls the unusual spermatogenesis, which is characterised by the exclusive formation of X-bearing sperm; (2) gene (*r*) encodes the elimination factor that binds to the X chromosome in the zygote, causing its elimination; (3) gene (*g*) controls the imprinting process that occurs in the mother and protects the maternally inherited X chromosome from elimination in the zygote and the elimination of the entire maternal chromosome complement during spermatogenesis; (4) gene (*e*) encodes the maternal factor produced during oogenesis, which inactivates the elimination factor [r] in the zygote, thus governing the elimination of the paternal X chromosome. The study involves the emergence of genes (*e*), (*s*), and (*r*) in an XX/X0 population where gene (*g*), which causes chromosomal imprinting, is already fixed in the population; that is, (*g*) is homozygous. The number and types of evolutionary transitions to the monogenic state depend on the dominant or recessive characteristics of the newly emerging genes (*e*), (*s*), and (*r*). Among the putative sequences (*e s r*), (*e r s*), (*s e r*), (*s r e*), (*r e s*), (*r s e*), only the sequences (*e s r*), (*s r e*), and (*r s e*) transform a digenic into a monogenic population. It was also found that the transition from a digenic population to a monogenic one does not occur in the absence of imprinting, so that the gene (*g*) responsible for the imprinting process must already be present in homozygosis (fixed) in the digenic population from which the monogenic population evolves; otherwise, the population cannot reach the monogenic state. This does not necessarily mean that the original digenic population had an imprinting process for sex determination. It could well be that the original population had such a process involved in a different biological process, if it existed, and that it was co-opted when the (*e*), (*s*), and (*r*) genes began to emerge in the population. An alternative model that can also explain the transition from a digenic to a monogenic population was also analysed. This model does not consider the existence of gene (*r*) and considers that the gene (*e*) regulates the number of X chromosomes eliminated in the zygote, as in the previous model, but it has the ability to interact directly with the X chromosome in the zygote, causing its elimination.

The models analysed here are based on conditions of positive selection for emerging genes. It remains to be evaluated whether other evolutionary forces, acting alone and in the absence of selection, can facilitate the evolution of a monogenic mechanism of sex determination from a digenic one.

## Figures and Tables

**Table 1:** Symbols and their biological meaning.

Symbol	Biological Meaning
Zygote *XX Ee Ss Rr Gg*	*X E S R G* comes from the mother and *X e s r g* comes from the father
Zygote *X0 Ee Ss Rr Gg*	*X E S R G* comes from the mother and *0 e s r g* comes from the father
Zygote *g*(*X0 Ee Ss Rr gG*)	The male comes from a mother where imprinting occurs
(*e*)	Gene (*e*)
[e]	The [e] product
(e*)	Gene (e*)
[e*]	The [e*] product
(*r*)	Gene (*r*)
[r]	The [r] product
0[e]	The amount of [e] is 0
1[e]	The amount of [e] is 1
2[e]	The amount of [e] is 2
1[r]	The amount of [r] is 1
2[r]	The amount of [r] is 2
1[e]:1[r]	1 dose of factor [e] inactivates 1 dose of factor [r]
1[e*]:2[r]	1 dose of factor [e*] inactivates 2 doses of factor [r]
*s^+^*(*e*)*dom*	Gene (*e*) is dominant
*s^+^*(*e*)*rec*	Gene (*e*) is recessive
*s^+^*(*s*)*dom*	Gene (*s*) is dominant
*s^+^*(*s*)*rec*	Gene (*s*) is recessive
*s^+^*(*r*)*dom*	Gene (*r*) is dominant
*s^+^*(*r*)*rec*	Gene (*r*) is recessive
*s^+^*(*g*)*dom*	Gene (*g*) is dominant
*s^+^*(*g*)*rec*	Gene (*g*) is recessive
(*Xe*)	The X chromosome carries the gene *e*
(*Xe*)*_m_*	The X chromosome that carries the gene (*e*) comes from the mother
(*Xe*)*_P_*	The X chromosome that carries the gene (*e*) comes from the father
*Inv*(*Xe*)*_m_*	The X chromosome that has an inversion and carries the gene (*e*) comes from the mother
*Inv*(*Xe*)*_p_*	The X chromosome that has an inversion and carries the gene (*e*) comes from the father
(*e s r*), (*e r s*), (*s e r*), (*s r e*), (*r e s*), (*r s e*)	All putative sequences of emergence of genes (*e*), (*s*) and (*r*) in the population
(*e s r*) example	The gene (*e*) emerges first, followed by the gene (*s*) and finally the gene (*r*)
SR	Sex ratio (males/females)
*g*(*X0*)	It indicates that the male comes from a mother where the imprinting process works

## Data Availability

The data presented in this study are available in the article and Supplementary Materials.

## Supplementary Materials

The following supporting information can be downloaded at: https://www.mdpi.com/article/10.3390/genes16121478/s1. Text S1. Function of gene (*s*). 2-factor model. Maternal imprinting. Paternal chromosomes eliminated. Text S2. Function of genes (*r*) and (*e*). 2-factor model. Maternal imprinting. Paternal chromosomes eliminated. Text S3. Calculation of genotypes frequencies. Text S4. Function of gene (*e*). 1-factor model. Maternal imprinting. Paternal chromosomes eliminated.
