# Supplementary material for: From Digenic to Monogenic Sex Determination in Insects: A Genetic Model Based on Imprinting and X Chromosome Elimination
Source: Genes (Basel). 2025 Dec 9;16(12):1478. doi: 10.3390/genes16121478 (PMC12732547; doi:10.3390/genes16121478)
Supplement: Supplementary file 1 [file genes-16-01478-s001.zip › genes-3846881-supplementary.pdf]

Text S1. Function of gene (*s*). 2-factor model. Maternal imprinting. Paternal chromosomes eliminated.

The gene (*s*) would be responsible for aberrant (AB) spermatogenesis, characterised by the segregation of chromosome sets inherited from both mother and father. That is, while in normal spermatogenesis (as occurs in female), the maternal and paternal chromosomes mix in the final sperm, in AB spermatogenesis, these chromosomes do not mix, but remain separate. Due to the process of maternal imprinting, only the genes inherited from the mother are transmitted to the sperm. The following sperm will be produced depending on the dominant or recessive nature of the gene (*s*).

(*s*) is dominant

Male	Sperm
g(X0 Ee Ss Rr gg)	*X E S R g (1)*
g(X0 eE sS rR gg)	*X e s r g (1)*

(*s*) is recesive

Male	Sperm
g(X0 Ee Ss Rr gg)	*X E S R g* (1/16)
	X e S R g (1/16)
	X E s R g (1/16)
	X E S r g (1/16)
	X e s R g (1/16)
	X e S r g (1/16)
	X E s r g (1/16)
	X e s r g (1/16)
	0 E S R g (1/16)
	0 e S R g (1/16)
	0 E s R g (1/16)
	0 E S r g (1/16)
	0 e s R g (1/16)
	0 e S r g (1/16)
	0 E s r g (1/16)
	0 e s r g (1/16)
**Male**	**Sperm**
g(X0 Ee ss Rr gg)	*X E s R g (1/4)*
g(X0 eE ss Rr gg)	*X e s R g (1/4)*
g(X0 Ee ss rR gg)	*X E s r g (1/4)*
g(X0 eE ss rR gg)	*X e s r g (1/4)*

Text S2. Function of genes (*r*) and (*e*). 2-factor model. Maternal imprinting. Paternal chromosomes eliminated.

The gene (*r*) encodes the elimination factor [r] that binds to the X chromosome causing its elimination. The gene (*e*) encodes the maternal factor [e], which interacts with the elimination factor [r] causing its inactivation. The stoichiometry of this interaction is considered to be 1[e]:1[r].

(*r*) is dominant

**Female**	**Oocyte**	**Male**	**Sperm**	**Zyote**	**Adult**
XX EE SS Rr gg	*X E S R g (1/2)*	*g(X0 EE SS RR gg)*	*X E S R g (1/2)*	*XX EE SS RR gg (1/4)*	*XX EE SS RR gg (1/4)*
	X E S r g (1/2)		*X E S R g (1/2)*	*XX EE SS rR gg (1/4)*	*g(X0 EE SS rR gg) (1/4)*
	X E S R g (1/2)		*0 E S R g (1/2)*	*X0 EE SS RR gg (1/4)*	*g(X0 EE SS RR gg) (1/4)*
	X E S r g (1/2)		*0 E S R g (1/2)*	*X0 EE SS rR gg (1/4)*	*g(X0 EE SS rR gg) (1/4)*

The zygote *XX EE SS rR gg* develops into the adult male *g(X0 EE SS rR gg)* because the mother does not carry the gene (*e*), which encodes the maternal product [e] that inactivates the [r] product. Consequently, this latter product interacts with the father-derived X chromosome causing its elimination. However, [r] cannot interact with the chromosome of maternal origin because it is imprinted; that is, it is resistant to the action of [r].

(*r*) is recessive

**Female**	**Oocyte**	**Male**	**Sperm**	**Zyote**	**Adult**
XX EE SS Rr gg	*X E S R g (1/2)*	*g(X0 EE SS RR gg)*	*X E S R g (1/2)*	*XX EE SS RR gg (1/4)*	*XX EE SS RR gg (1/4)*
	X E S r g (1/2)		*X E S R g (1/2)*	*XX EE SS rR gg (1/4)*	*XX EE SS rR gg (1/4)*
	X E S R g (1/2)		*0 E S R g (1/2)*	*X0 EE SS RR gg (1/4)*	*g(X0 EE SS RR gg) (1/4)*
	X E S r g (1/2)		*0 E S R g (1/2)*	*X0 EE SS rR gg (1/4)*	*g(X0 EE SS rR gg) (1/4)*

The zygote *XX EE SS rR gg* develops into the adult female *XX EE SS rR g* because the gene (*r*) is recessive; that is, the gene (*r*) needs to be in homozygosis to perform its function (interact with the X chromosome causing its elimination.

**Female**	**Oocyte**	**Male**	**Sperm**	**Zyote**	**Adult**
XX ee SS Rr gg	*X e S R g (1/2)*	*g(X0 ee SS rr gg)*	*X e S r g (1/2)*	*XX ee SS Rr gg (1/4)*	*XX ee SS Rr gg (1/4)*
	X e S r g (1/2)		*X e S r g (1/2)*	*XX ee SS rr gg (1/4)*	*XX ee SS rr gg (1/4)*
	X e S R g (1/2)		*0 e S r g (1/2)*	*X0 ee SS Rr gg (1/4)*	*g(X0 ee SS Rr gg) (1/4)*
	X e S r g (1/2)		*0 e S r g (1/2)*	*X0 ee SS rr gg (1/4)*	*g(X0 ee SS rr gg) (1/4)*

The XX ee SS rr gg zygote develops into an adult female despite carrying two doses of the [r] elimination factor, since the mother is homozygous for the (*e*) gene and, consequently, produces oocytes with two doses of factor 2[e] that inactivates the [r] factor. Only the paternally inherited X chromosome is eliminated, not the maternally inherited one, since the latter is imprinted; that is, it is resistant to the interaction of the [r] factor.

Text S3. Calculation of genotypes frequencies.

Relative frequency of female (XX)

$$F_{n+1}(XX) = \frac{\{\Sigma[F_n(XX)\times F_n(X0)\times F(G_{XX})]\times(SV_{XX})\}}{\Sigma F_{n+1}\ allfemales\ genotypes}$$

Relative frequency of male $(X0)$

$$F_{n+1}(X0) = \frac{\{\Sigma[F_n(XX)\times F_n(X0)\times F(G_{X0})]\times(SV_{X0})\}}{\Sigma F_{n+1} all\ males\ genotypes}$$

$F_n(XX)$ = Relative frequency of female genotype (XX) at generation (n)

$F_n(X0)$ = Relative Frequency of male genotype $(X0)$ at generation (n)

$F(G_{XX})$ = Frequency of females with genotype *(XX)* produced by the particular cross $F_n(XX)\times F_n(X0)$

$F(G_{X0})$= Frequency of males with genotype *(X0)* produced by the particular cross $F_n(XX)\times F_{\square}(X0)$

$F_{n+1}(XX)$ = Relative frequency of female genotype (XX) at generation $(n+1)$

$F_{n+1}(X0)$ = Relative frequency of male genotype $(X0)$ at generation $(n+1)$

(SV_{XX}) and (SV_{X0}) refer, respectively, to the selective value for female of genotype *(XX)* and for male of genotype *(X0)*. $(SV_{XX}) = (1-\omega_{XX})$ and $(SV_{X0}) = (1-\omega_{X0})$, where ω_{XX} and ω_{X0} refer to the selection coefficient of XX female and X0 male, respectively. It is considered that ω is not sex-specific so that $\omega_{XX} = \omega_{X0}$

$\{(SV_{XX}) = (XV_{X0})\} = \{(SV_e), (SV_s), (SV_r), (SV_{es}), (SV_{er}), (SV_{sr}), (SV_{esr})\}$, where $(SV_e), (SV_s), (SV_r), (SV_{es}), (SV_{er}), (SV_{sr}), (SV_{esr})$ represent the normalised additive values of genotypes carrying different combinations of the new genes (e), (s) and (r):

$(SV_e) = (1-\omega_e)$

$(SV_s) = (1-\omega_s)$

$(SV_r) = (1 - \omega_r)$

$(SV_{es}) = [(1 - \omega_e) + (1 - \omega_s)]/2$

$(SV_{er}) = [(1 - \omega_e) + (1 - \omega_r)]/2$

$(SV_{sr}) = [(1 - \omega_s) + (1 - \omega_r)]/2$

$(SV_{esr}) = [(1 - \omega_e) + (1 - \omega_s) + (1 - \omega_r)]/3$

Genotype frequencies are a set of recurrent equations whose values in each generation depend on the frequencies of all existing genotypes in the previous generation. Simulations are run until a steady state is reached, defined by the constant frequency of the genotypes that make up the population. In all scenarios, the initial state of the population corresponds to a digenic XX/X0 population in which one of the new genotypes has emerged with a frequency of 0.1 or 0.01 (in the case of the emergence of a new genetic system corresponding to a monogenic population, the initial frequency of the new genotypes of 0.001 was also tested). The calculations were carried out with Microsoft Excel.

Text S4. Function of gene (*e*). 1-factor model. Maternal imprinting. Paternal chromosomes eliminated.
The gene (*e*) is expressed in the mother during oogenesis, and its product accumulates in the oocyte. The gene (*e*) controls the number of X chromosomes eliminated in the zygote by interacting with the paternally derived X chromosome, resulting in its elimination; the stoichiometry of the interaction is 1[e]:1[X]. Factor [e] cannot interact with the maternally derived chromosome because it is protected by imprinting.

(*s*) is recessive

**Female**	**Oocyte**	**Male**	**Sperm**	**Zyote**	**Adult**
XX EE Ss gg	*X E S g* (1/2)	*g(X0 eE ss gg)*	*X e s g* (1)	*XX Ee Ss gg* (1)	*XX Ee Ss gg* (1)
	X E s g (1/2)		*X e s g* (1)	*XX Ee ss gg* (1/2)	*XX Ee ss gg* (1/2)

The female does not carry the gene (*e*). Therefore, the zygote does not carry the maternal factor 1[e]. Consequently, X chromosome elimination does not occur.

**Female**	**Oocyte**	**Male**	**Sperm**	**Zyote**	**Adult**
XX ee Ss gg	*X e S g* (1/2)	*g(X0 eE ss gg)*	*X e s g* (1)	*XX ee Ss gg* (1/2)	g(*X0 ee Ss gg*) (1/2)
	X e s g (1/2)		*X e s g* (1)	*XX ee ss gg* (1/2)	*g(X0 ee ss gg*) (1/2)

The female carries two doses of the gene (*e*). Therefore, the zygote carries maternal factor 2[e]. Consequently, the paternally derived X chromosome is eliminated, while the maternally derived X chromosome is resistant to interaction with factor [e] because it is imprinted.
